# Conductance of ‘bare-bones’ tripodal molecular wires†

**DOI:** 10.1039/c8ra01257a

**Published:** 2018-06-28

**Authors:** Ross J. Davidson, David C. Milan, Oday A. Al-Owaedi, Ali K. Ismael, Richard J. Nichols, Simon J. Higgins, Colin J. Lambert, Dmitry S. Yufit, Andrew Beeby

**Affiliations:** Department of Chemistry, Durham University South Rd Durham DH1 3LE UK ross.davidson@durham.ac.uk andrew.beeby@durham.ac.uk; Department of Chemistry, University of Liverpool Crown St Liverpool L69 7ZD UK nichols@liv.ac.uk; Department of Physics, University of Lancaster Lancaster LA1 4YB UK; Department of Laser Physics, Women Faculty of Science, Babylon University Hilla Iraq; Department of Physics, College of Education for Pure Science, Tikrit University Tikrit Iraq

## Abstract

Controlling the orientation of molecular conductors on the electrode surfaces is a critical factor in the development of single-molecule conductors. In the current study, we used the scanning tunnelling microscopy-based break junction (STM-BJ) technique to explore ‘bare-bones’ tripodal molecular wires, employing different anchor groups (AGs) at the ‘top’ and ‘bottom’ of the tripod. The triarylphosphine tris(4-(methylthio)phenyl)phosphine and its corresponding phosphine sulfide showed only a single high conductance feature in the resulting 1- and 2-dimensional conductance histograms, whereas analogous molecules with fewer than three thiomethyl AGs did not show clear conductance features. Thus, by systematic molecular modifications and with the aid of supporting DFT calculations, the binding geometry, with respect to the surface, was elucidated.

## Abstract 2

When venturing into the field of tripodal molecular conductors the geometry of the tripod with respect to the surface is a critical factor affecting conductance. Here we examine the behaviour of a tripodal conductor by systematic modifications of a triarylphosphine system.

## Graphical abstract

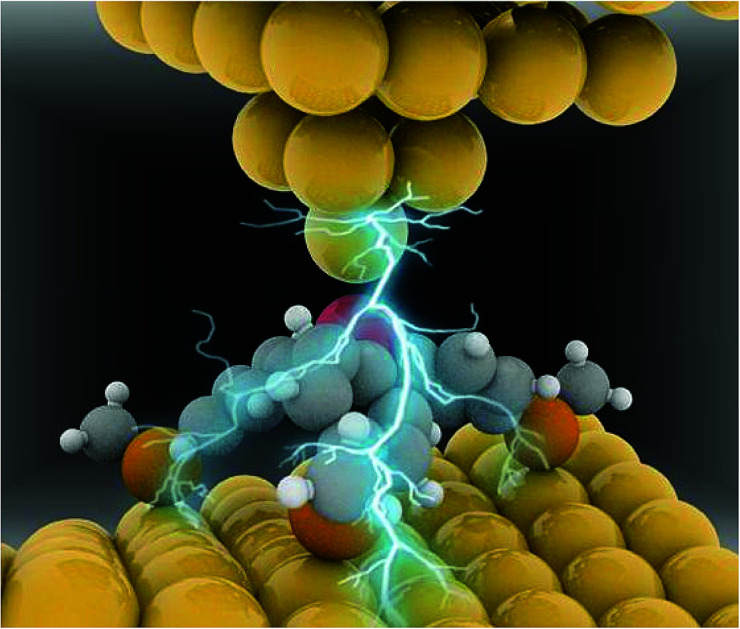

## Introduction

The field of single molecule conductance has almost exclusively involved the study of one-dimensional molecular wires, with one anchor group (AG), *e.g.* pyridine, thiol or amine, attached at each end.^1,2^ This motif has been favoured as it provides the least-complicated example for examining structure-property relationships and conductance mechanisms. However, to afford greater spatial control relative to the substrate it is necessary to increase the number of AGs to give tripodal conductors. Increasing the number of AGs significantly enhances the strength of attachment of the molecule to the surface(s), thereby increasing the mechanical stability of the metal|molecule|metal junction.^3,4^ Recent studies demonstrated the use of tripodal molecules consisting of three monodentate AGs (*e.g.*, pyridine, thiophene) attached to a central carbon or silicon atom, bridged by a conductor such as phenylene or phenylene ethynylene, to give conductance values of 2 × 10^−5^ and 5 ± 1 × 10^−4^*G*_0_ respectively.^3,5,6^ However, in each reported example, two or more AGs were attached to the central conjugated unit *via* sp^3^ hybridised carbon or silicon atoms (by the use of a carbon atom, spirofluorene, or adamantane motif),^5,7–9^ reducing the degree of conjugation between each of the AGs.

To examine the electrical conductance of a tripodal molecular wire in its simplest form, we examined a ‘bare-bones’ structure consisting a triaryl phosphine with AGs on each of the aryl groups. Ragaini demonstrated that 4,4′,4′′-phosphanetriyltribenzenethiol can simultaneously coordinate to metal complexes such as Ni(CO)_3_*via* the phosphine, and chemisorb to a gold surface *via* the thiols to give a trigonal pyramidal structure on the surface.^10^

Parameswaran *et al.* has previously demonstrated that diphenylphosphine moieties can function as mechanically and chemically stable AGs with a tendency to bind to under-coordinated gold atoms on the surface.^11^ In addition to enhanced stability compared with corresponding dialkylphosphines, diphenylphosphines couple well to the σ-system of alkanes resulting in relatively high conductances for Ph_2_P(CH_2_)_*n*_PPh_2_ junctions. Additionally, phosphines can also be readily oxidised by sulfur to give the corresponding phosphine sulfide. Fukazara demonstrated that dibenzophospholesulfides act as strong AGs and when bridged by 1,4-phenylene and biphenyl-4,4′-diyl groups, give conductance values of 5 × 10^−4^ and 5 × 10^−5^*G*_0_ respectively and favour conductance *via* the LUMO.^12^

Here we report conductance measurements and calculations on ‘bare-bones’ tripodal molecular wires based on triaryl-phosphines, -phosphine sulfides, -phosphine oxides and -selenides using scanning tunnelling microscopy (STM)-based techniques and we examine how conductance is affected by the number of available AGs in the tripods making contact with the single apex terminal group. The triarylphosphines and their P(v) derivatives are a good test case for this due to their simplicity and the fact that they can be easily modified in a modular fashion.

## Experimental methods

### Synthetic work

Compound 3 was prepared by the literature methods,^10^ the synthesis of compounds 1, 2, 1–3=S, 1–3=Se, 2=O, 3=O, and [3-Me]^+^ are given in the ESI.†

### Conductance measurements

All measurements were performed at room temperature and under ambient conditions with an Agilent (Keysight) STM running Picoscan 5.3.3. software. The STM-BJ setup used to perform all the measurements consists of a home-made scanner^13^ controlled by an Agilent Picoscan 5500 controller with a N9503A modified scanner and a multichannel current amplifier. Samples were prepared by adsorption onto commercially-obtained gold-on-glass Au(111) substrates prepared with a chromium adhesion layer (Arrandee). The gold slides were flame annealed with a butane torch. The process involved heating the gold-on-glass slide in a dark room until it shows an orange glow, then allowing the slide to cool; this process is repeated as many times as necessary to obtain the desired Au(111) flat surface.^14^ Sample solutions were prepared by dissolving the amount of compound (a few milligrams) in 10 mL of mesitylene needed to achieve a 10^−4^ M concentration. Gold STM tips were fabricated from 0.25 mm Au wire (99.99%) that was freshly mechanically cut for each STM experiment. Conductance experiment were carried out adding 100 μL of the 10^−4^ M solution into the STM-BJ liquid cell, then the STM-BJ tip was repeatedly approached into contact and then withdrawn from the Au(111) surface. A current set point of 100 μA was used to achieve a good gold contact with the surface, and the tip was then retracted 4 nm away from the surface at a rate of 20 nm s^−1^. A bias voltage of 100 mV was applied between the sample and tip; current–distance traces were recorded during the approach to contact and the withdrawal process.

## Results and discussion

### Synthesis

**Chart 1:**
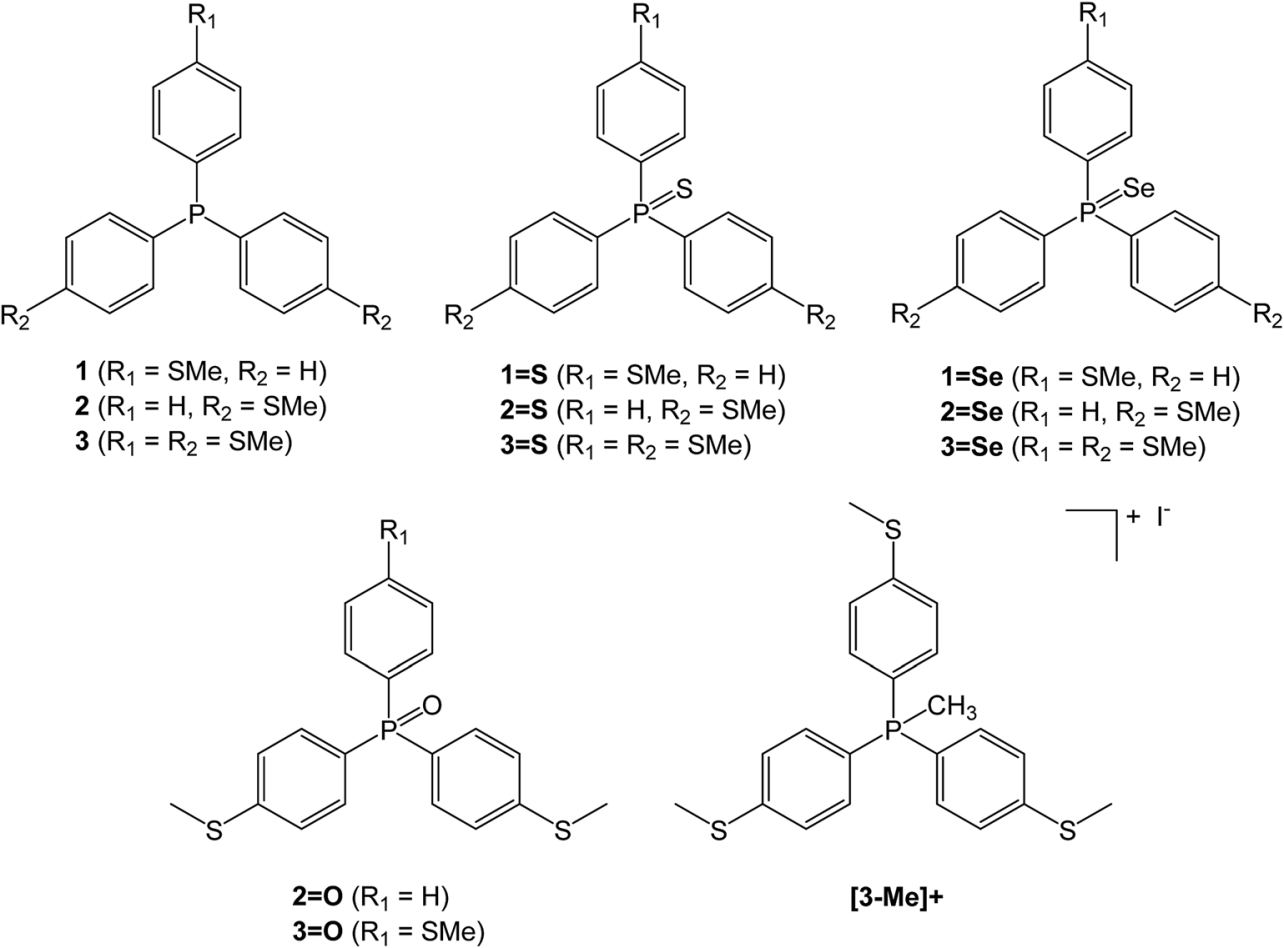
Triaryl phosphines and their derivatives used in this investigation.

A family of triaryl phosphines and their oxidised derivatives were chosen as the tripods with aryl groups consisting of combinations of either thioanisole or phenyl groups. Thiomethyl AGs have been chosen due to their chemical stability and their proven record as contact groups in molecular junctions.^15^ Each of the phosphines were synthesised by carrying out halogen-lithium exchange on (4-bromophenyl)(methyl)sulfane, followed by reaction with PCl_3_, dichlorophenylphosphine or chlorodiphenylphosphine.^10,16^ The phosphines (1–3) were then heated in the presence of either sulfur (to produce the respective phosphine sulfides 1=S, 2=S and 3=S) or selenium (to produce the phosphine selenides 1=Se, 2=Se and 3=Se). The phosphine oxides 2=O and 3=O were prepared by reaction of (4-(methylthio)phenyl)lithium with phenylphosphinic dichloride or phosphoryl bromide respectively, to avoid complications from oxidation of thiomethyl groups that arose upon attempted H_2_O_2_ oxidation of 2 and 3. To aid control experiments (*q.v.*), the methylphosphonium iodide salt of 3 ([3-Me]^+^) was synthesised using the standard techniques of reacting 3 with methyl iodide (Chart 1).

### Molecular conductance

**Fig. 1:**
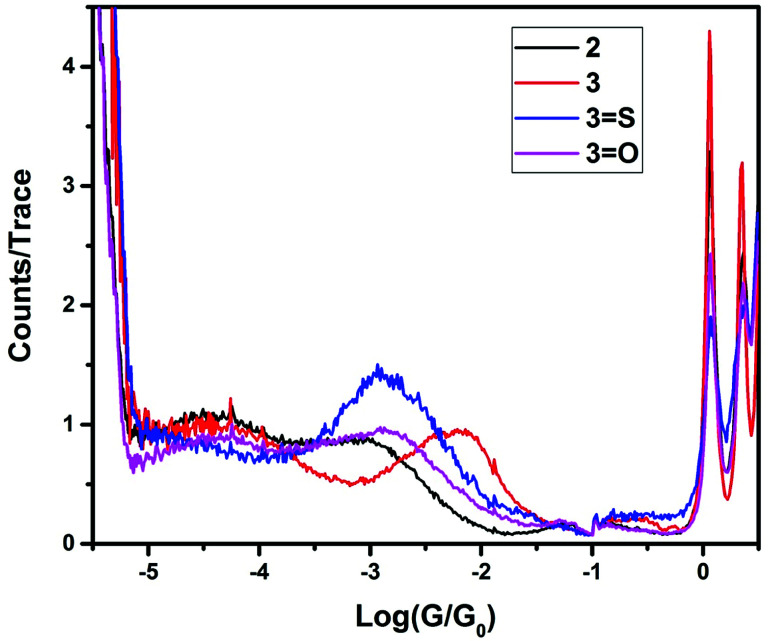
Conductance histograms for the compounds 2, 3, 3=S and 3=O.

The single-molecule conductances of tripodal compounds were determined using the STM-BJ method.^17^ Conductance values and break-off distances (95th percentile) are summarized in Table 1. Conductance histograms for the compounds (2, 3, 3=S, and 3=O) are shown in Fig. 1. Representative conductance traces with current plateaus, two-dimensional (2D) histograms; conductance values and break-off distances (95th percentile) are shown in the ESI.†

**Table 1:** Conductance values and break-off distances (95th percentile) for 2, 3, 3=S and 3=O

Molecule	Conductance (*G*_0_)	Break-off distance (nm)
1	No peak	
2	3.46 × 10^−5^	0.76
	5.88 × 10^−4^	0.94
3	4.36 × 10^−3^	0.76
1=S	No peak	
2=S	No peak	
3=S	1.23 × 10^−3^	0.87
3=O	4.46 × 10^−5^	0.91
	1.09 × 10^−3^	0.76
2=O	No peak	
[3-Me]^+^	No peak	

Each of the three phosphine selenide compounds (1=Se–3=Se) showed a complete absence of molecular conductance peaks. In addition, in experiments with the phosphine selenides, the *G*_0_ peaks in the histograms were significantly altered, with much-diminished peak heights, indicating that the gold surface had been chemically altered. The most likely cause of this alteration is ‘selenium poisoning’ resulting from the surface-induced decomposition of the phosphine selenide. In fact, in the present study, we examined the stability of these compounds by a ^1^H and ^31^P NMR study in CDCl_3_; see ESI.† This analysis shows that the compounds decayed over a number of hours, primarily to the corresponding phosphine oxide liberating the selenium.

In contrast, the 3 and 3=S compounds showed single, well-defined conductance peaks, at 10^−2.36^ and 10^−2.91^*G*_0_ respectively, similar in value to 1,4-bis(methylthio)benzene (10^−2.15^*G*_0_).^18^ However, replacement of a single thiomethyl group with a hydrogen resulted in two conductance peaks being observed for compound 2 (10^−3.23^ and 10^−4.46^*G*_0_), and no conductance peaks being observed for compound 2=S.

Further replacement of the thiomethyl groups with hydrogens (*i.e.*, 1 and 1=S) resulted in no molecular conductance peak being observed, suggesting that the conductance path is not simply that between one single thiomethyl group and the central donor group (either phosphorus or phosphorus sulfide). To further elucidate the nature of the central atom involvement in junction formation, we performed STM-BJ studies on the corresponding phosphine oxides (compounds 2=O and 3=O) and on the methylphosphonium iodide compound [3-Me]I. Compound 3=O displayed conductance peaks at 10^−2.96^ and 10^−4.35^, while compound 2=O displayed no conductance peak, as for the sulfide 2=S. The presence of a second peak for 3=O suggests that the phosphine oxide does not bind to the surface as strongly as the sulfide, allowing the formation of other contact geometries, possibly consisting of a thiomethyl-molecule-thiomethyl contacted arrangement based on the increased break-off distance for the low conductance band. Finally, [3-Me]^+^ showed no detectable conductance peak, which suggests that junction formation *via* thiomethyl groups at each contact does not contribute significantly to the conductance peaks in these molecules. Rather, for the highest conductance mode to be reached, the central atom (P or P=S) must be able to act as a contact for one electrode, and all three thiomethyl groups must be available to make contact with the surface, giving the tripodal shape of the molecule. Indeed, previous work by Ragaini demonstrated that on a similar compound (4,4′,4′′-phosphanetriyltribenzenethiol),^10^ contact occurs *via* the aryl thiols leaving the central donor phosphorus atom available to act as a binding site able to coordinate to metal carbonyls.

To account for the low conductance mode that occurs when only two thiomethyl groups are attached, we propose a model in which one P–C_6_H_4_–SMe lies flat on the gold surface, allowing the lone pair in conjunction with the π-orbitals of the thioanisole to make contact with the surface. This arrangement would leave a single thioanisole raised perpendicular to the surface, providing a contact for the second electrode. In fact, similar behaviour has been observed by Su *et al.*,^19^ whereby the 1,1-bis(4-(methylthio)phenyl)siletane system displayed two conductance peaks, which were an order of magnitude different. The low conductance peak corresponded to the end-to-end thiomethyl–thiomethyl contact mode, while the high conductance peak corresponded to attachment formed between the thiomethyl group at one gold contact and the central silicon at the other gold contact.

### Theoretical calculations

Using the density functional code SIESTA^4^ the optimum geometries of the isolated molecules were obtained by relaxing the molecules until all forces on the atoms were less than 0.05 eV Å^−1^. A double-zeta plus polarization orbital basis set,^20,21^ norm-conserving pseudopotentials, an energy cut-off of 250 Rydbergs defined the real space grid. Transmission functions were computed using and the local density approximation (LDA),^22^ a generalized gradient approximations (GGA),^23^ and using a van der Waals^24^ functional and were found to be qualitatively similar in all cases,^25^ (see Fig. S45 in the ESI†).

The use of DFT to compute the ground state energy of various molecular junctions permits us to calculate binding energies to gold electrodes and optimal geometries. To avoid basis set superposition errors (BSSE)^26^ we use the counterpoise correction^27^ in which the binding energy of a molecule denoted a to an electrode b is expressed as:1
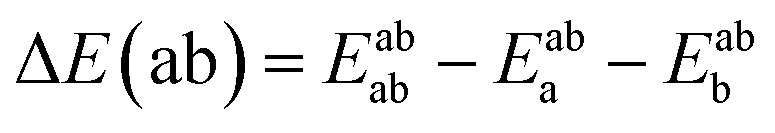


In this expression, the total energy of the combined a and b system is 
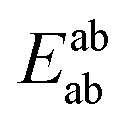
, while the total energies of isolated systems a and b are 
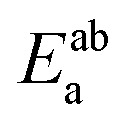
 and 
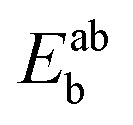
 respectively, maintaining identical basis sets (a,b) for the three energies.

**Fig. 2:**
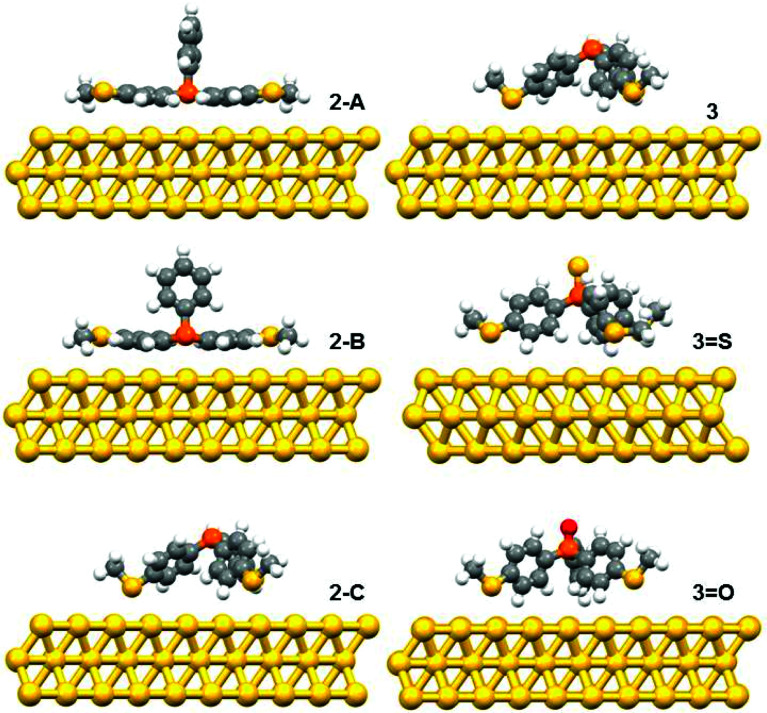
The relaxed geometries of all possible structures for 2, 3, 3=S and 3=O; where atoms are indicated by grey (carbon), white (hydrogen), yellow (sulfur), red (oxygen) and orange (phosphorus).

**Fig. 3:**
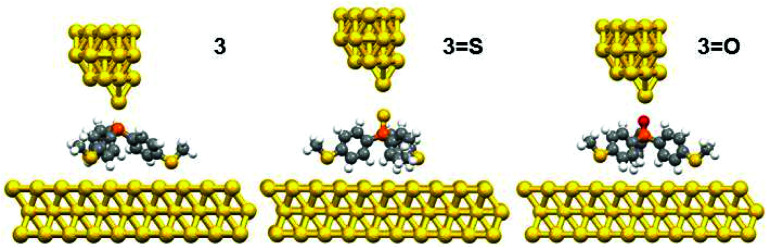
The relaxed geometries of all molecular junctions; where atoms are indicated by grey (carbon), white (hydrogen), yellow (sulfur), red (oxygen) and orange (phosphorus).

The DFT calculations predicted three possible geometries for the system involving two thiomethyl AGs (compound 2). Based on the binding energy calculations shown in Table 2, the most probable geometry was shown to be 2-C (Fig. 2), since the order of binding energies between the molecule and gold electrode follows the trend |*E*_2-C_| > |*E*_2-B_| > |*E*_2-A_|. For the structures with three thiomethyl anchor groups, our model shows one possible geometry for compounds 3, 3=S and 3=O (Fig. 3). The highest binding energy value is presented by compound 3 (Δ*E* = −1.02 eV).

**Table 2:** The binding energies Δ*E*(ab) of molecules to the Au(111) surface, calculated using a vdW functional. For comparison the corresponding binding energies obtained using GGA are shown. To obtain these results, we started from 252 different initial conditions for each molecule and allowed each to fully relax to a minimum energy. Then from these 252 different simulations, we chose the relaxed structure corresponding to the lowest energy. Fig. S41–S44 in the ESI show the various local energy minima obtained from this procedure

System	Δ*E*(ab) (eV) GGA	Δ*E*(ab) (eV) vdW
2-A	−0.57	−0.61
2-B	−0.76	−0.79
2-C	−0.82	−0.85
3	−0.94	−1.02

To further explain the conductance behaviour we employed DFT-based transport code GOLLUM^28^ to compute the transmission coefficient *T*(*E*) for each relaxed junction geometry (see Fig. 3). This was achieved by first obtaining the corresponding Hamiltonian and overlap matrices with SIESTA, using a double-zeta polarized basis set. The optimized junction geometries as shown in Fig. 3 confirm well that the thiomethyl-contacted compounds are not oriented normal to the electrode surface within the molecular junction. Rather, they are tilted within molecular junctions to accommodate the directionality of the lone pairs of electrons on the sulfur atoms that bind to the gold electrodes.^3,4^ From *T*(*E*), the electrical conductance *G* was obtained using the Landauer formula.

**Fig. 4:**
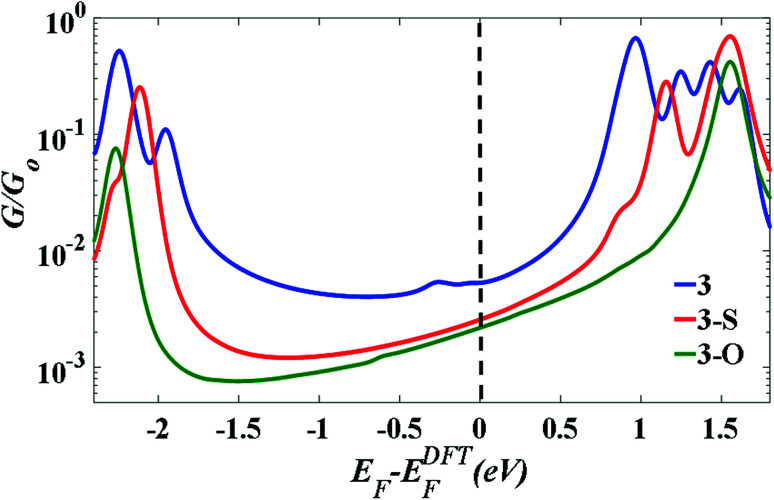
The calculated conductance as a function of Fermi energy for all molecular junctions, with molecules bound to Au (111) surfaces, obtained using a van der Waals functional.^20,21^

Fig. 4 indicates that in all cases the Fermi level lies near the centre of the HOMO–LUMO gap, but shifted slightly towards the LUMO resonance, and therefore a LUMO-mediated electron tunnelling mechanism is anticipated. These results are consistent with our previous studies.^1,2^ The order of the calculated conductances is *G*_3_ > *G*_3-S_ > *G*_3-O_ (Table 3).

**Table 3:** The experimental (Exp. *G*/*G*_0_) and calculated conductance values (Th. *G*/*G*_0_) at *E*_F_ − 
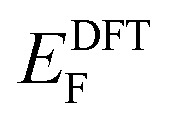
 = 0 eV. Z* is the experimental break-off distance. The calculated electrode separation in relaxed junctions (*Z*); *Z* = *d*_Au–Au_ − 0.25 nm, where 0.25 nm is the calculated centre-to-centre distance of the apex atoms of the two opposing gold electrodes when conductance = *G*_0_ in the absence of a molecule. *d*_Au–Au_ is the calculated centre-to-centre distance of the apex atoms of the two opposing gold electrodes in relaxed junctions

Molecule	Exp. *G*/*G*_0_	Th. *G*/*G*_0_	Z* (nm)	*Z* (nm)	*d*_Au–Au_ (nm)
3	4.36 × 10^−3^	5.5 × 10^−3^	0.76	0.27	0.52
3-S	1.23 × 10^−3^	2.8 × 10^−3^	0.87	0.39	0.64
3-O	1.09 × 10^−3^	2.5 × 10^−3^	0.76	0.26	0.51

## Conclusions

In conclusion we have investigated the molecular conductance of a ‘bare-bones’ tripodal structure, consisting of either tris(4-(methylthio)phenyl)phosphine (3) or phosphine sulfide (3=S), and with both the thiomethyl and phosphorus/phosphine sulfide acting as surface binding groups. Using STM-BJ, we determined that both 3 and 3=S are highly conductive, with only a single conductance peak.

Through the systematic substitution of thiomethyl groups with hydrogens, it was determined that three thiomethyl groups were required to achieve the single conductance mode, inferring that molecules bind to the surface *via* the thiomethyl groups, leaving the phosphorus (3) or phosphine sulfide (3=S) available to act as a ‘top’ contact for the molecular bridge. This was supported by DFT calculations, which showed that this geometry was the only stable geometry for the 3, 3=S and 3=O. The calculations also indicate the Fermi-energy level lies in the vicinity of the middle of the HOMO–LUMO gap slightly closer to the LUMO.

## Conflicts of interest

The authors declare no competing financial interest.

## Supplementary Material

RA-008-C8RA01257A-s001

RA-008-C8RA01257A-s002

RA-008-C8RA01257A-s003
