# Supplementary material for: Conductance of ‘bare-bones’ tripodal molecular wires
Source: RSC Adv. 2018 Jun 28;8(42):23585–90. doi: 10.1039/c8ra01257a (PMC9081744; doi:10.1039/c8ra01257a)
Supplement: RA-008-C8RA01257A-s001 [file RA-008-C8RA01257A-s001.pdf]

Electronic Supplementary Material (ESI) for RSC Advances.
This journal is © The Royal Society of Chemistry 2018

Supplementary Information

Conductance of 'bare-bones' tripodal molecular wires

Ross J. Davidson,[a*] David C. Milan,[b] Oday A. Al-Owaedi,[c,d] Ali K. Ismael,[c,e] Richard J. Nichols,[b*] Simon J. Higgins,[b] Colin J. Lambert,[c] Dmitry S. Yufit,[a] Andrew Beeby,[a*].

[a]*Department of Chemistry, Durham University, South Rd, Durham, DH1 3LE, UK*

[b]*Department of Chemistry, University of Liverpool, Crown St, Liverpool, L69 7ZD, UK*

[c]*Department of Physics, University of Lancaster, Lancaster LA1 4YB, U.K.*

[d]*Department of Laser Physics, Women Faculty of Science, Babylon University, Hilla, Iraq.*

[e]*Department of Physics, College of Education for Pure Science, Tikrit University, Tikrit, Iraq*

Table of Contents

a) Experimental

1. Synthesis S3
2. NMR spectra of reported compounds S11
3. Selenide decay S28
4. Crystallographic data S30

b) Conductance measurements

1. Conductance 2D Histograms S35
2. Conductance 1D Histograms S36
3. Representative conductance traces S37

c) Theoretical details

1. Geometry of isolated molecules S38
2. Binding energy of **2 (A, B,** and **C)** and **3** on gold S39
3. Conductance S43

d) References S44

a) Experimental

Instrumentation. Microanalyses were performed by Elemental Analysis Service, London Metropolitan University, UK. NMR spectra were recorded on a Bruker Avance (^{1}H 400.13, ^{13}C 100.61 MHz) ASAP data were recorded on a Xevo QTOF (Waters) high resolution, accurate mass tandem mass spectrometer equipped with Atmospheric Pressure Gas Chromatography (APGC) and Atmospheric Solids Analysis Probe (ASAP). Listed peaks correspond to the most abundant isotopmer; assignments were made by a comparison of observed and simulated spectra.

General details. All chemicals were purchased from standard chemical suppliers without further purification. tris(4-(methylthio)phenyl)phosphane (**3**) was synthesised according to literature methods.[1]

1. Synthesis.

(4-(methylthio)phenyl)diphenylphosphine (**1**). n-Butyllithium (9.84 mL, 2.5 M, 24.6 mmol) was slowly added to a solution containing 4-bromothioanisole (5.00 g, 24.6 mmol) at -78°C under an inert atmosphere, the solution was maintained at this temperature and stirred for a further hour before chlorodiphenylphosphine (4.53 mL, 5.41 g, 24.6 mmol) was added dropwise, the solution was maintained at -78°C for an hour before the solution was allowed to warm to room temperature, the solution was stirred for a further 12 hours before the solution was poured into a $NH_4Cl_{(aq)}$ solution, the solution was washed with DCM, the organic layer was collected and dried with $MgSO_4$. Hexane was added to the DCM solution and allowed to evaporate slowly forming large white crystals. Yield: 3.33 g (44 %). ^{1}H NMR (CD_2Cl_2) δ: 7.37-7.35 (m, 3H), 7.34-7.31 (m, 7H), 7.26-7.22 (m, 4H), 2.48 (s, 3H). ^{13}C NMR (CD_2Cl_2) δ: 140.1, 137.4, 137.4 (d, J_{CP} = 11 Hz), 134.1 (d, J_{CP} = 20 Hz), 133.5 (d, J_{CP} = 20 Hz), 133.1 (d, J_{CP} = 11 Hz), 133.0, 128.6, 128.4 (d, J_{CP} = 7 Hz), 125.8 (d, J_{CP} = 7 Hz), 14.9

ppm. ^{31}P NMR (CD_2Cl_2) δ: -6.54 (s) ppm. ASAP: m/z 309.079 $[M+H]^+$. Anal. Calc. for $C_{19}H_{17}PS$: C, 74.00; H, 5.56 %. Found: C, 73.86; H, 5.49 %.

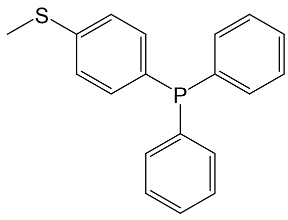

bis(4-(methylthio)phenyl)(phenyl)phosphine (**2**). The same procedure as for **1** except $PPhCl_2$ was used in place of PPh_2Cl. Yield: 2.43 g (28 %) ^1H NMR (CD_2Cl_2) δ: 7.36-7.34 (m, 3H), 7.32-7.29 (m, 2H), 7.24-7.21 (m, 8H), 2.48 (s, 6H) ppm. ^{13}C NMR (CD_2Cl_2) δ: 140.0, 134.0 (d, J_{CP} = 11 Hz), 133.9 (d, J_{CP} = 20 Hz), 133.4 (d, J_{CP} = 20 Hz), 133.1 (d, J_{CP} = 11 Hz), 128.6, 128.4 (d, J_{CP} = 7 Hz), 125.8 (d, J_{CP} = 7 Hz), 14.9 ppm. ^{31}P NMR (CD_2Cl_2) δ: -7.47 (s) ppm. ASAP: m/z 355.061 $[M+H]^+$. Anal. Calc. for $C_{20}H_{19}PS_2$: C, 67.77; H, 5.40 %. Found: C, 67.48; H, 5.50 %.

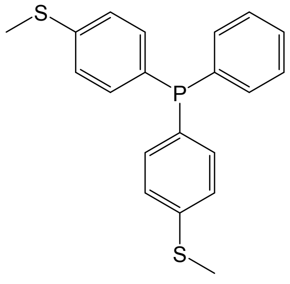

General Phosphine Sulfide synthesis. Phosphine (1 eq) was added to a solution containing sulfur (5 eq) in toluene (30 mL). The solution was degassed with three freeze-pump-thaw cycles before the solution was heated to reflux for 1 hour, after which the solvent was removed. The remaining solid was dissolved in a minimal amount of dichloromethane before

hexane was added until a white precipitate formed; the precipitate was collected by filtration to give a white powder, the desired product.

(4-(methylthio)phenyl)diphenylphosphine sulfide (**1=S**). Yield: 378 mg (89 %). ^{1}H NMR (CD_2Cl_2) δ: 7.74-7.71 (m, 4H), 7.64-7.60 (m, 2H), 7.56-7.53 (m, 2H), 7.49-7.45 (m, 4H), 7.30 (dd, J = 8, 3 Hz, 2H), 2.50 (s, 3H) ppm. ^{13}C NMR (CD_2Cl_2) δ: 144.3 (d, J_{CP} = 3 Hz), 133.3, 132.9, 132.4 (d, J_{CP} = 11 Hz), 132.0 (d, J_{CP} = 11 Hz), 131.5 (d, J_{CP} = 3 Hz), 128.5, 128.4, 125.2, 125.1, 14.6 ppm. ^{31}P NMR (CD_2Cl_2) δ: 42.4 (s) ppm. ASAP: m/z 341.043 [M+H]$^+$. Anal. Calc. for $C_{19}H_{12}PS_2$: C, 67.03; H, 5.03 %. Found: C, 66.91; H, 5.01 %.

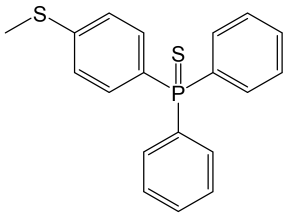

bis(4-(methylthio)phenyl)(phenyl)phosphine sulfide (**S=2**). Yield: 390 mg (81 %). ^{1}H NMR (CD_2Cl_2) δ: 7.72-7.69 (m, 2H), 7.61-7.58 (m, 4H), 7.55-7.52 (m, 1H), 7.46 (dd, J = 8, 5 Hz), 2H), 7.29 (dd , J = 8, 2 Hz, 4H), 2.50 (s, 6H) ppm. ^{13}C NMR (CD_2Cl_2) δ: 144.2 (d, J_{CP} = 3 Hz), 132.2 (d, J_{CP} = 85 Hz), 132.3 (d, J_{CP} = 11 Hz), 132.0 (d, J_{CP} = 11 Hz), 131.5 (d, J_{CP} = 3 Hz), 128.7-128.2 (m), 125.1 (d, J_{CP} = 13 Hz), 14.6 ppm. ^{31}P NMR (CD_2Cl_2) δ: 41.96 (s) ppm. ASAP: m/z 387.082 [M+H]$^+$. Anal. Calc. for $C_{20}H_{19}PS_3$: C, 62.15; H, 4.95 %. Found: C, 61.84; H, 4.93 %.

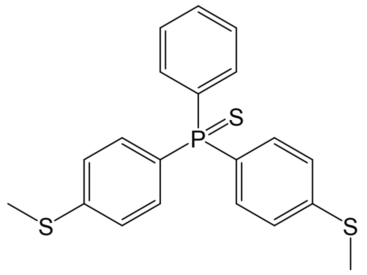

tris(4-(methylthio)phenyl)phosphine sulfide (**3=S**). Crystals were gown by the evapouration of a DCM/Hexane solution. Yield: 489 mg (90 %). ^{1}H NMR (CD_2Cl_2) δ: 7.63-7.58 (m, 6H), 7.29-7.26 (m, 6H), 2.50 (s, 9H) ppm. ^{13}C NMR (CD_2Cl_2) δ: 145.1, 144.0, 132.3-132.2 (m), 125.3 (d, J_{CP} = 13 Hz), 14.7 ppm. ^{31}P NMR (CD_2Cl_2) δ: 41.7 ppm. ASAP: m/z 433.017 $[M+H]^+$. Anal. Calc. for $C_{21}H_{21}PS_4$: C, 58.30; H, 4.89 %. Found: C, 58.33; H, 4.91 %.

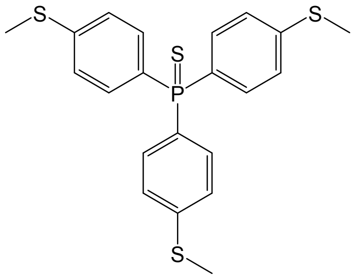

General Phosphine Selenide synthesis. Phosphine (1 eq) was added to a solution containing selenium (5 eq) in toluene (30 mL). The solution was degassed with three freeze-pump-thaw cycles before the solution was heated to reflux for 16 hours, after which the solution was filtered and the solvent was removed from the filtrate. The remaining solid was dissolved in a minimal amount of dichloromethane before hexane was added until a white precipitate formed; the precipitate was collected by filtration to give a white powder, the desired product.

(4-(methylthio)phenyl)diphenylphosphine selenide (**1=Se**). Yield: 363 (75 %). ^{1}H NMR (CD_2Cl_2) δ: 7.75-7.72 (m, 4H), 7.65-7.61 (m, 2H), 7.54-7.52 (m, 2H), 7.49-7.45 (m, 4H), 7.29 (dd, J = 8, 3 Hz, 2H), 2.50 (s, 3H) ppm. ^{13}C NMR (CD_2Cl_2) δ: 144.4 (d, J_{CP} = 3 Hz), 132.7 (d, J_{CP} = 11 Hz), 132.4 (d, J_{CP} = 11 Hz), 132.2, 131.7, 131.6 (d, J_{CP} = 3 Hz), 128.5 (d, J_{CP} = 12 Hz), 125.1 (d, J_{CP} = 13 Hz), 14.6 ppm. ^{31}P NMR (CD_2Cl_2) δ: 34.4 (s) ppm. ASAP: m/z 388.996 $[M+H]^+$. Anal. Calc. for $C_{19}H_{17}PSSe$: C, 58.92; H, 4.42 %. Found: C, 59.03; H, 4.51 %.

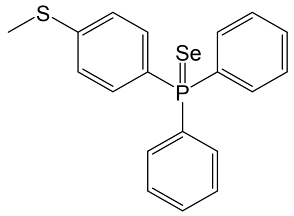

bis(4-(methylthio)phenyl)(phenyl)phosphine selenide (**Se=2**). Yield: 81 mg (15 %). ^{1}H NMR (CD_2Cl_2) δ: 7.72-7.69 (m, 2H), 7.62-7.58 (m, 4H), 7.54-7.51(m, 1H), 7.47-7.44 (m, 2H), 7.28 (dd, J = 8, 2 Hz, 4H), 2.50 (s, 6H) ppm. ^{13}C NMR (CD_2Cl_2) δ: 144.4 (d, J_{CP} = 3 Hz), 132.7 (d, J_{CP} = 11 Hz), 132.3 (d, J_{CP} = 11 Hz), 131.5 (d, J_{CP} = 3 Hz), 128.5 (d, J_{CP} = 13 Hz), 127.4, 127.0, 125.1 (d, J_{CP} = 13 Hz), 14.6 ppm. ^{31}P NMR (CD_2Cl_2) δ: 33.70 (s) ppm. ASAP: m/z 434.981 $[M+H]^+$. Too unstable for elemental analysis.

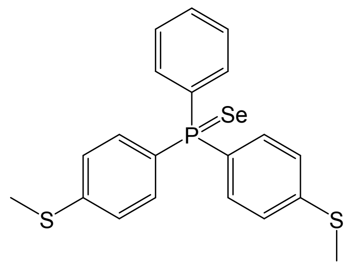

tris(4-(methylthio)phenyl)phosphine selenide (**3=Se**). Crystals were gown by the evapouration of a DCM/Hexane solution. Yield: 486 mg (81 %). ^{1}H NMR (CD_2Cl_2) δ: 7.60-7.57 (m, 2H), 7.53-7.50 (m, 4H), 7.31-7.27 (m, 6H), 2.50 (s, 9H) ppm. ^{13}C NMR (CD_2Cl_2) δ: 144.3, 132.6 (d, J_{CP} = 12 Hz), 132.0 (d, J_{CP} = 11 Hz), 125.1-125.0 (m), 14.5 ppm. ^{31}P NMR (CD_2Cl_2) δ: 32.9 ppm (s). ASAP: m/z 480.969 $[M+H]^+$. Anal. Calc. for $C_{21}H_{21}PS_4$: C, 52.6; H, 4.41 %. Found: C, 52.98; H, 4.42 %.

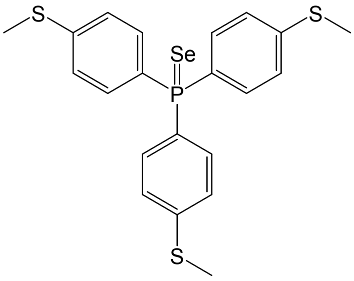

tris(4-(methylthio)phenyl)phosphine oxide (**3=O**). THF (100 mL, dry) was added to 4-bromothioanasole (5.00 g, 25 mmol), the solution was cooled to -78 °C before nBuLi (2.5 M, 10 mL, 25 mmol) was added slowly. The solution was stirred at -78 °C for 2 hours before $POBr_3$ (0.85 mL, 2.38 g, 8.33 mmol) was added dropwise added, the temperature was maintained at -78°C for an additional hour before being warmed to room temperature and stirred overnight. After 16 hours the reaction was quenched with water and extracted with dichoromethane. The organic layer was dried over $MgSO_4$ before the solvent was removed. The residual oil was triturated with hexane three times before it was dissolved in DCM and precipitated with hexane, to yield a white solid. Crystals were grown by evapouration of a THF solution. Yield: 1.73 g (50 %). ^{1}H NMR (CD_2Cl_2) δ: 7.52 (dd, J = 11.5, 8.3 Hz, 6H), 7.30 (dd, J = 8.3, 2.0 Hz, 6H), 2.52 (s, 9H) ppm. ^{13}C NMR (CD_2Cl_2) δ: 144.4, 132.2 (d, J = 10.5 Hz), 128.4, 127.7, 125.3 (d, J = 12.5 Hz), 14.9 ppm. ^{31}P NMR (CD_2Cl_2) δ: 28.10 (s)

ppm. ASAP: m/z 417.053 $[M+H]^+$. Anal. Calc. for $C_{21}H_{21}PS_3O$: C, 62.97; H, 5.28 %. Found: C, 62.92; H, 5.47 %.

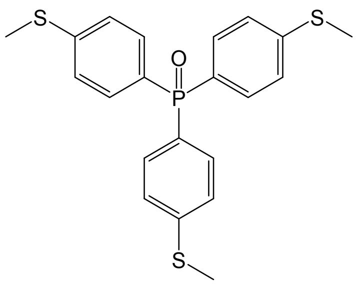

bis(4-(methylthio)phenyl)(phenyl)phosphine oxide (**O=2**). The same procedure as for **O=3** except phenylphosphinic dichloride was used in place of phosphoryl tribromide. Yield: 2.25 g (73 %). ^{1}H NMR (CD_2Cl_2) δ: 7.64-7.60 (m, 2H), 7.53-7.48 (m, 5H), 7.44-7.41 (m, 2H), 7.26-7.22 (m, 4H), 2.46 (s, 6H) ppm. ^{13}C NMR (CD_2Cl_2) δ: 144.4 (d, J_{CP} = 3 Hz), 132.3-132.1 (m), 132.0-131.9 (m), 128.4 (d, J_{CP} = 12 Hz),, 125.2 (d, J_{CP} = 12 Hz), 14.7 ppm. ^{31}P NMR (CD_2Cl_2) δ: 28.82 (s) ppm. ASAP: m/z 371.058 $[M+H]^+$. Anal. Calc. for $C_{20}H_{19}OPS_2$: C, 64.84; H, 5.17 %. Found: C, 64.90; H, 5.42 %

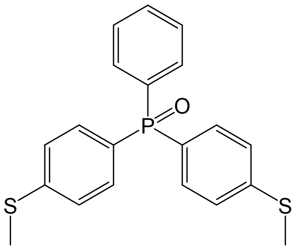

methyltris(4-(methylthio)phenyl)phosphonium iodide (**[3-Me]**$^+$). Iodomethane (46 µL, 105 mg, 1.50 mmol) was added to a solution containing 3 (300 mg, 0.75 mmol) in THF (20 mL), the solution was stirred for 12 hours forming a white precipitate. The precipitate was collected via filtration and washed with DCM to give a white powder. Yield: 373 mg, (85 %)

^{1}H NMR ($CDCl_3$) δ: 7.60-7.57 (m, 6H), 7.43 (dd, J = 8.5, 2.8 Hz, 6H), 3.10 (d, J = 13 Hz, 3H), 2.52 (s, 9H) ppm. ^{13}C NMR ($CDCl_3$) δ: 149.6 (d, J_{CP} = 3 Hz), 133.1 (d, J_{CP} = 13 Hz), 126.4 (d, J_{CP} = 13 Hz), 113.5 (d, J_{CP} = 94 Hz), 14.5, 11.7 (d, J_{CP} = 58 Hz) ppm. ^{31}P NMR ($CDCl_3$) δ: 20.3 (pseudo singlet) ppm. ASAP: m/z = 415.069 $[M-I]^{+}$. Too unstable for elemental analysis.

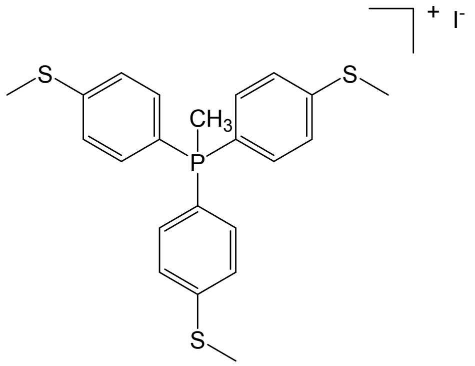

2. NMR spectra of reported compounds

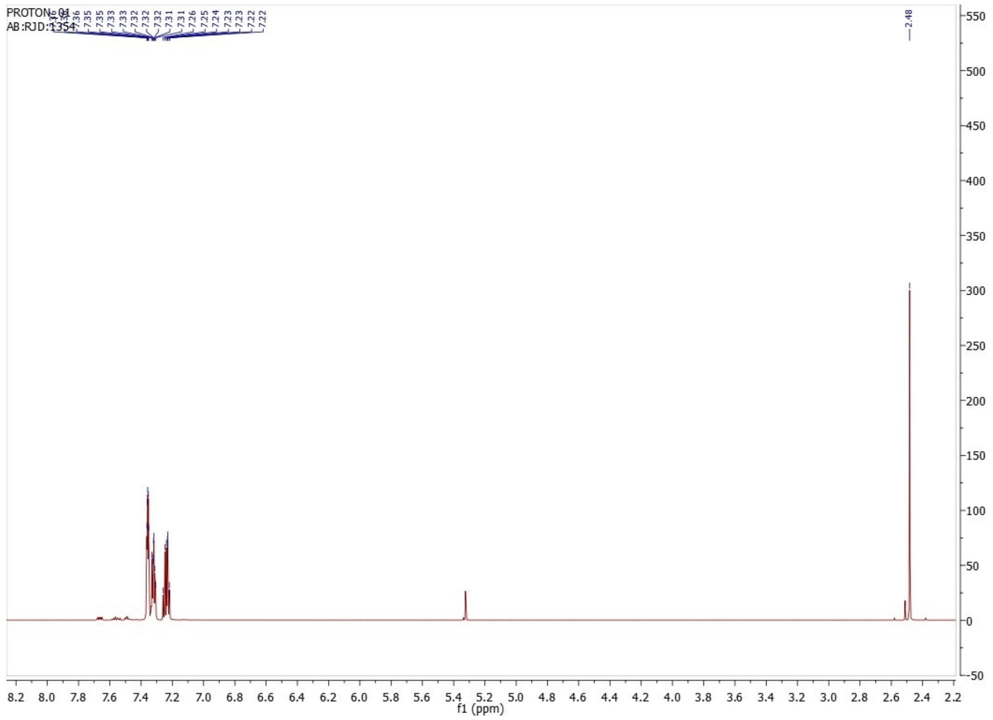

Figure S1. ^{1}H NMR of **1** recorded in CD_2Cl_2.

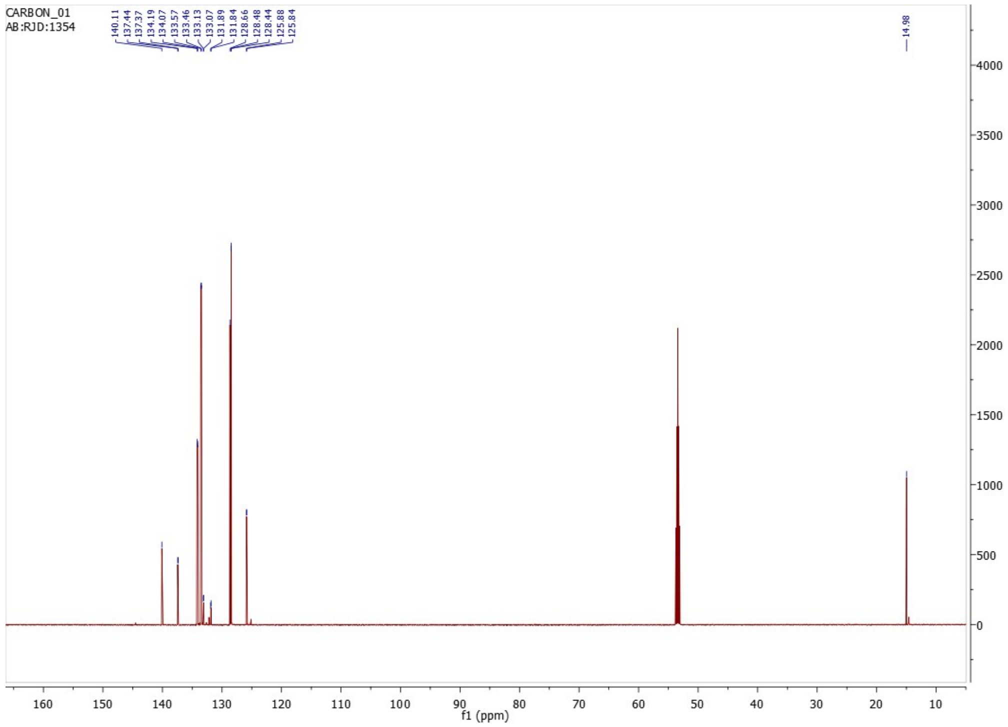

Figure S2. ^{13}C NMR of **1** recorded in CD_2Cl_2.

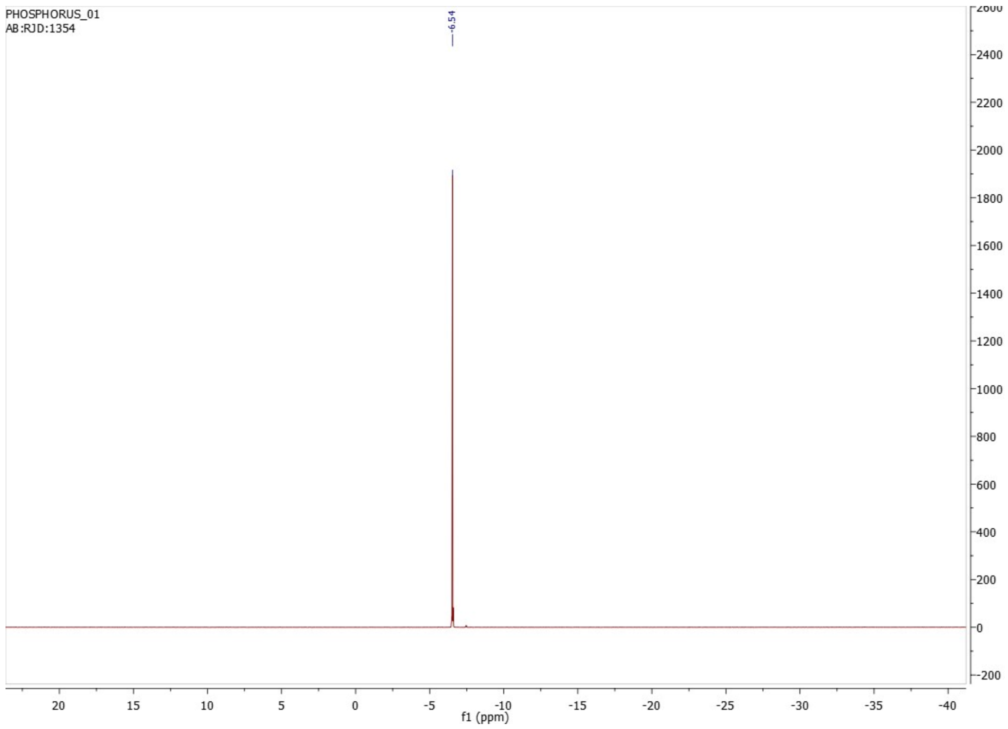

Figure S3. ^{31}P NMR of **1** recorded in CD_2Cl_2.

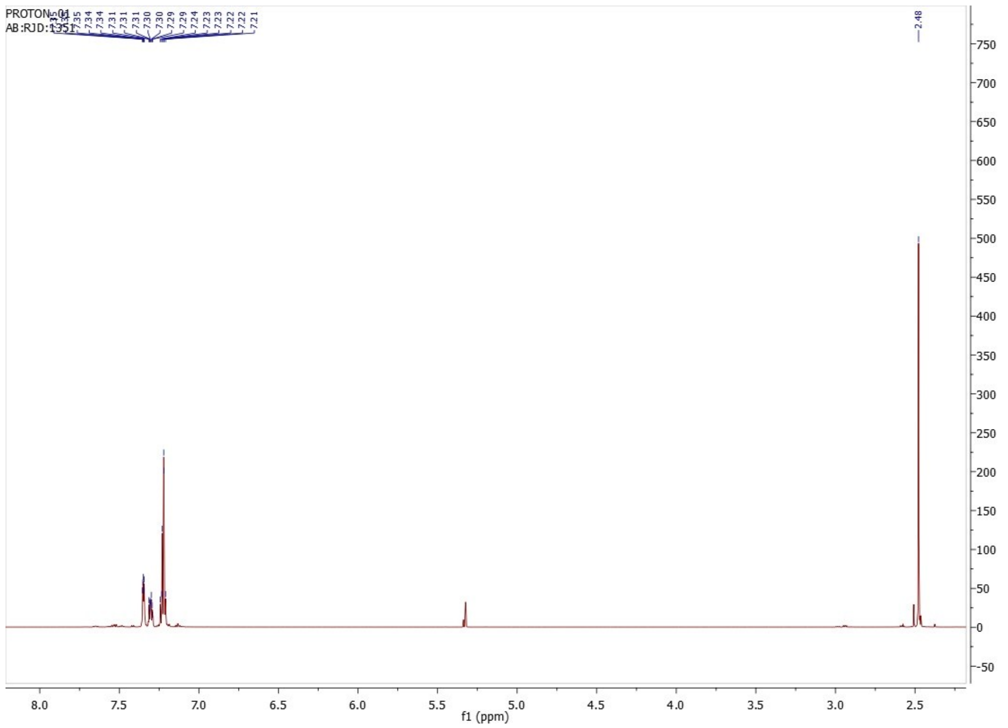

Figure S4. ^{1}H NMR of **2** recorded in CD_2Cl_2.

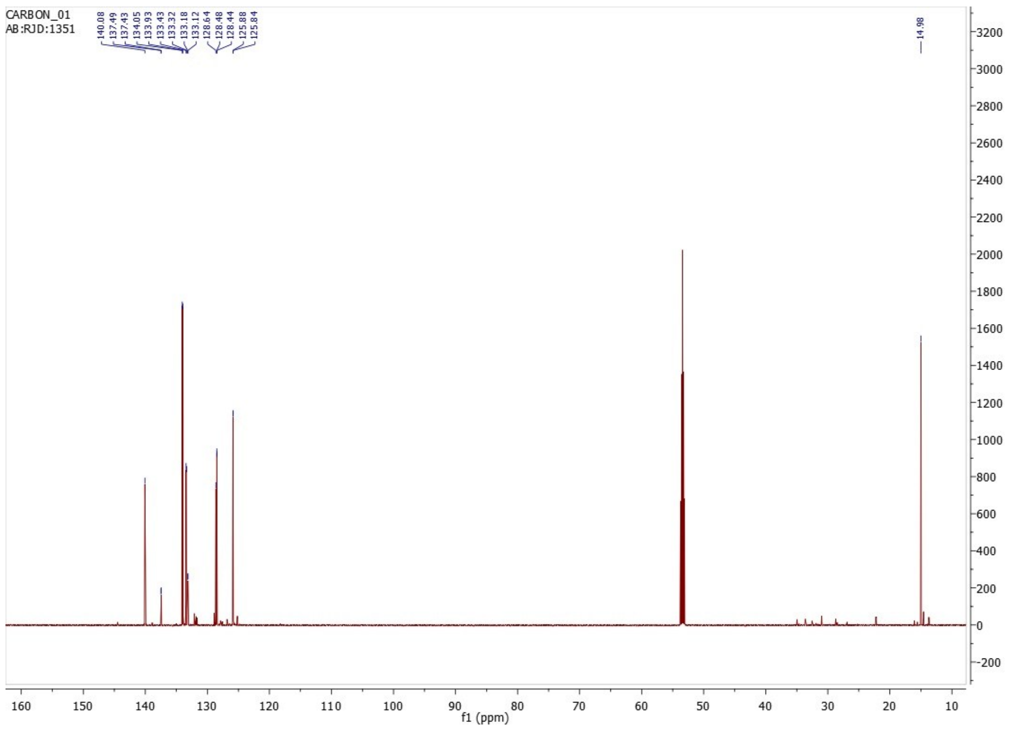

Figure S5. ^{13}C NMR of **2** recorded in CD_2Cl_2.

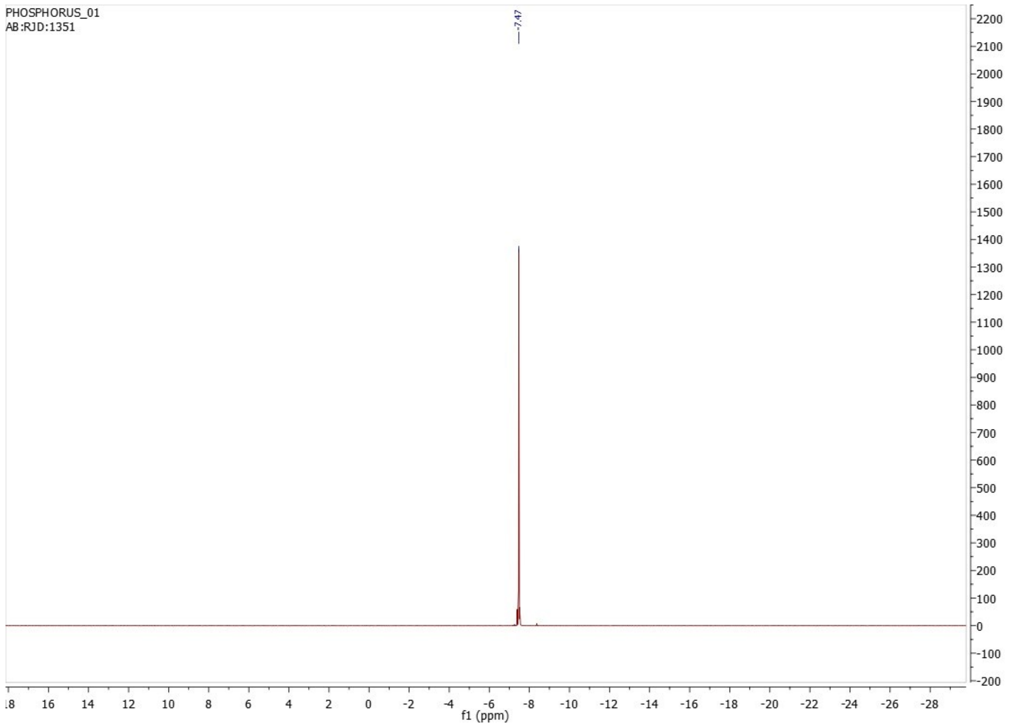

Figure S6. ^{31}P NMR of **2** recorded in CD_2Cl_2.

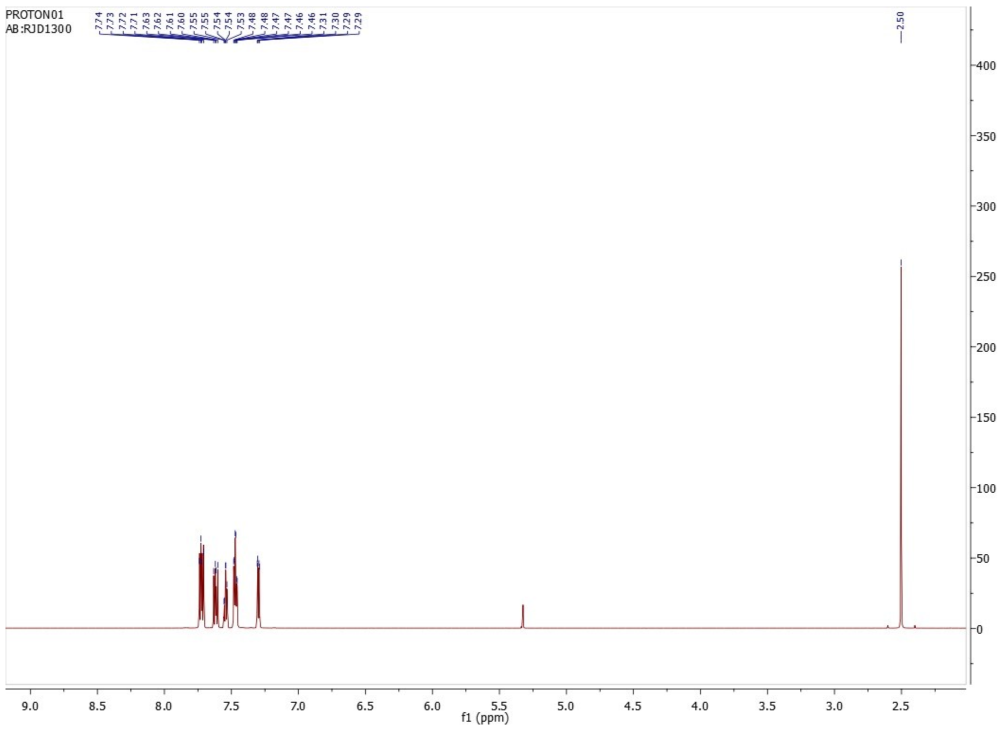

Figure S7. ^{1}H NMR of **1=S** recorded in CD_2Cl_2.

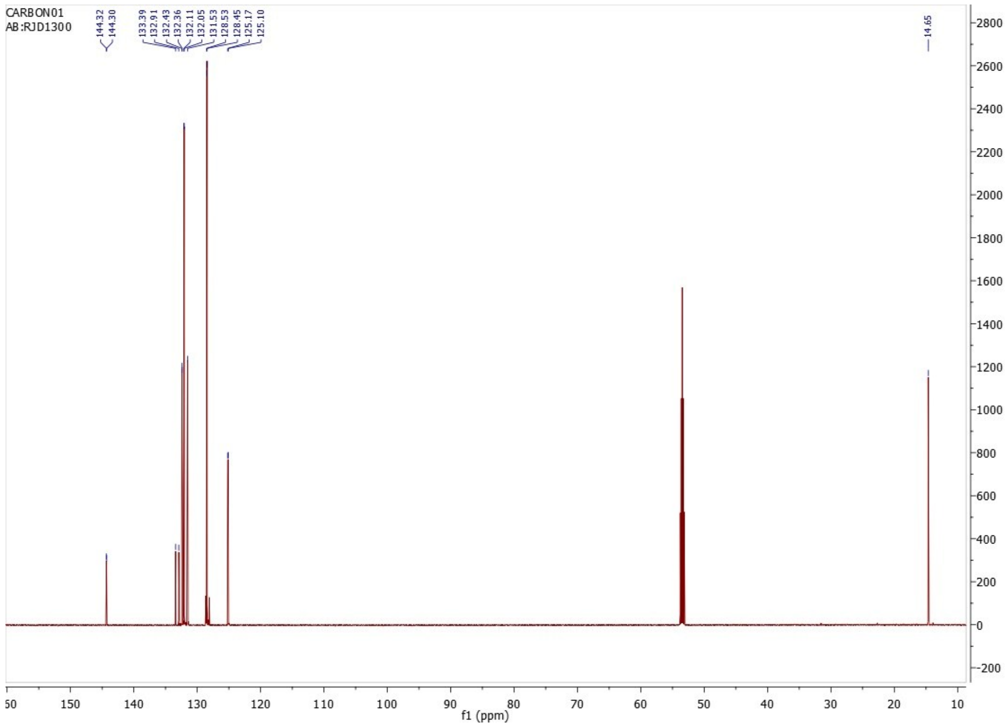

Figure S8. ^{13}C NMR of **1=S** recorded in CD_2Cl_2.

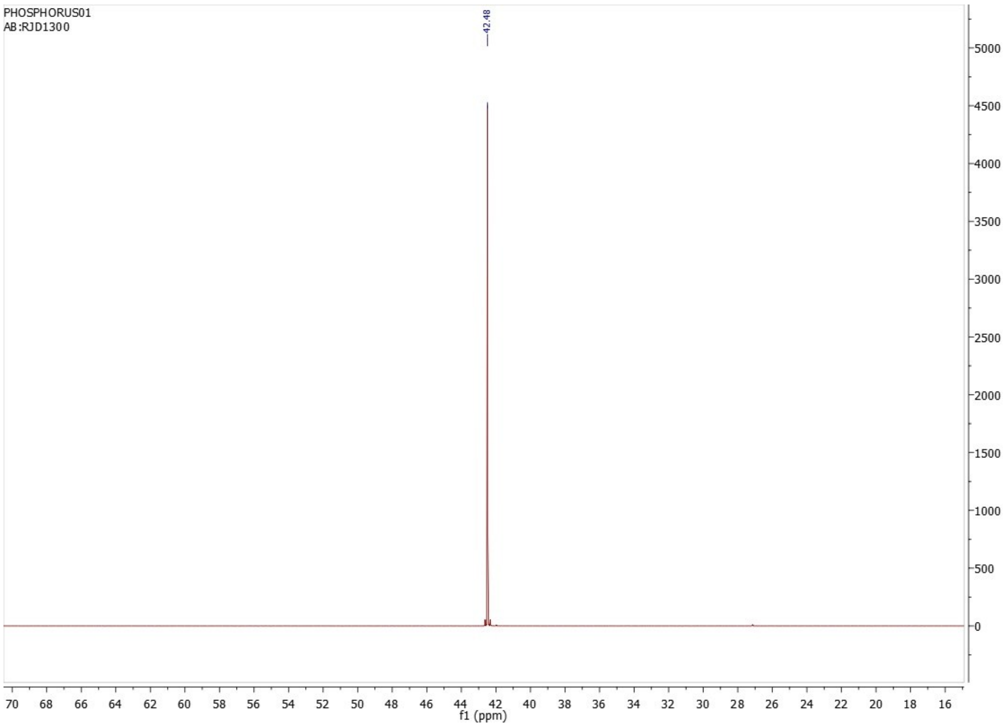

Figure S9. ^{31}P NMR of **1=S** recorded in CD_2Cl_2.

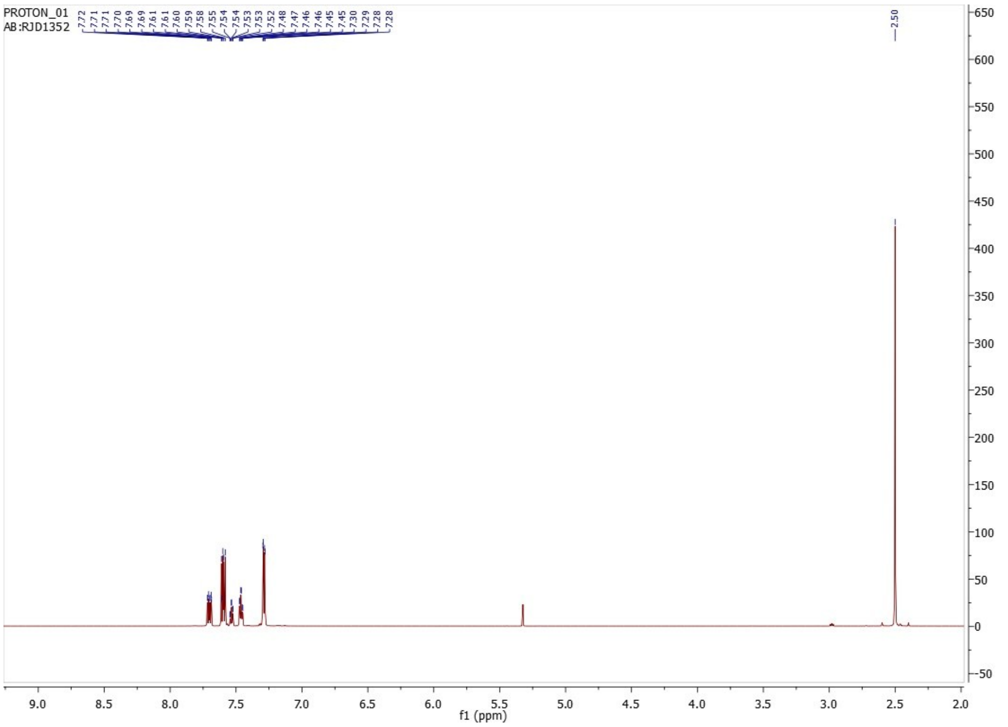

Figure S10. ^{1}H NMR of **2=S** recorded in CD_2Cl_2.

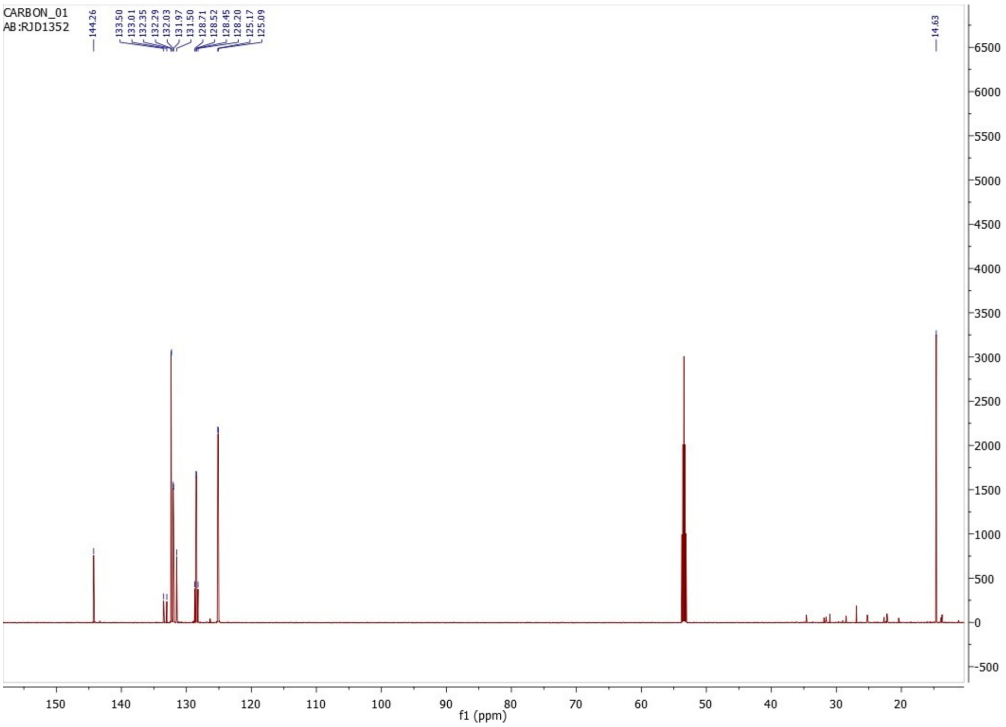

Figure S11. ^{13}C NMR of **2=S** recorded in CD_2Cl_2.

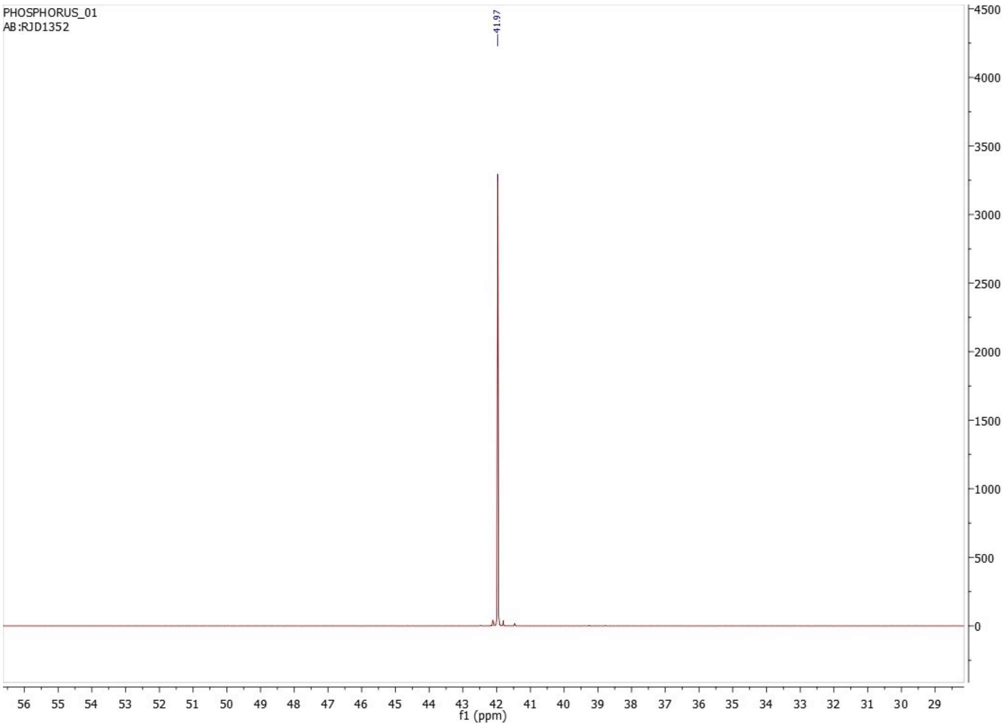

Figure S12. ^{31}P NMR of **2=S** recorded in CD_2Cl_2.

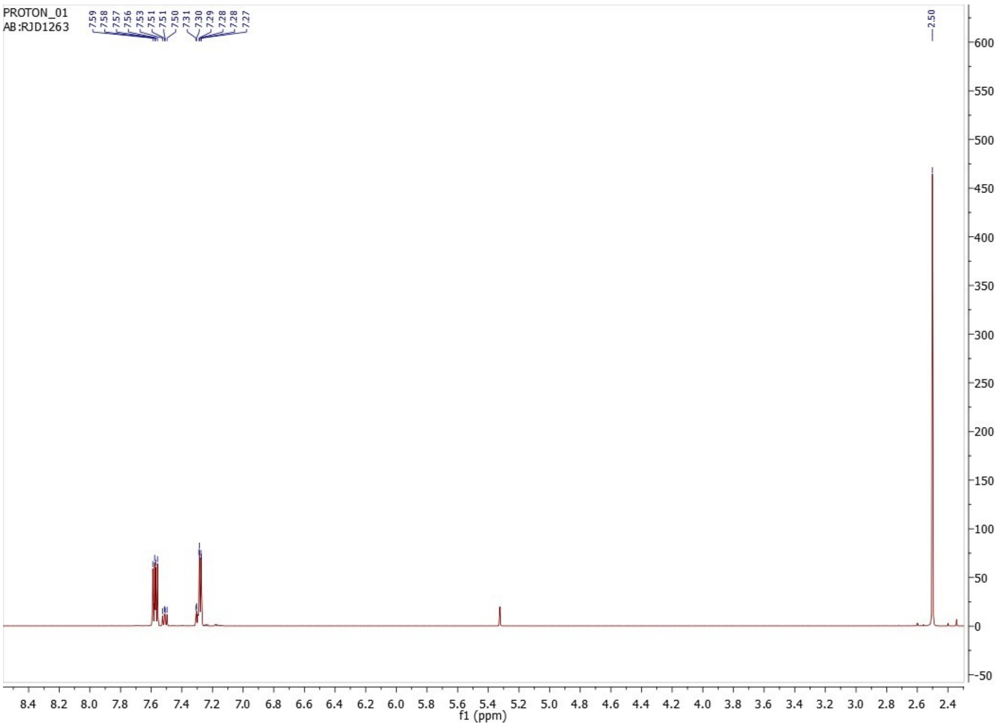

Figure S13. ^{1}H NMR of **3=S** recorded in CD_2Cl_2.

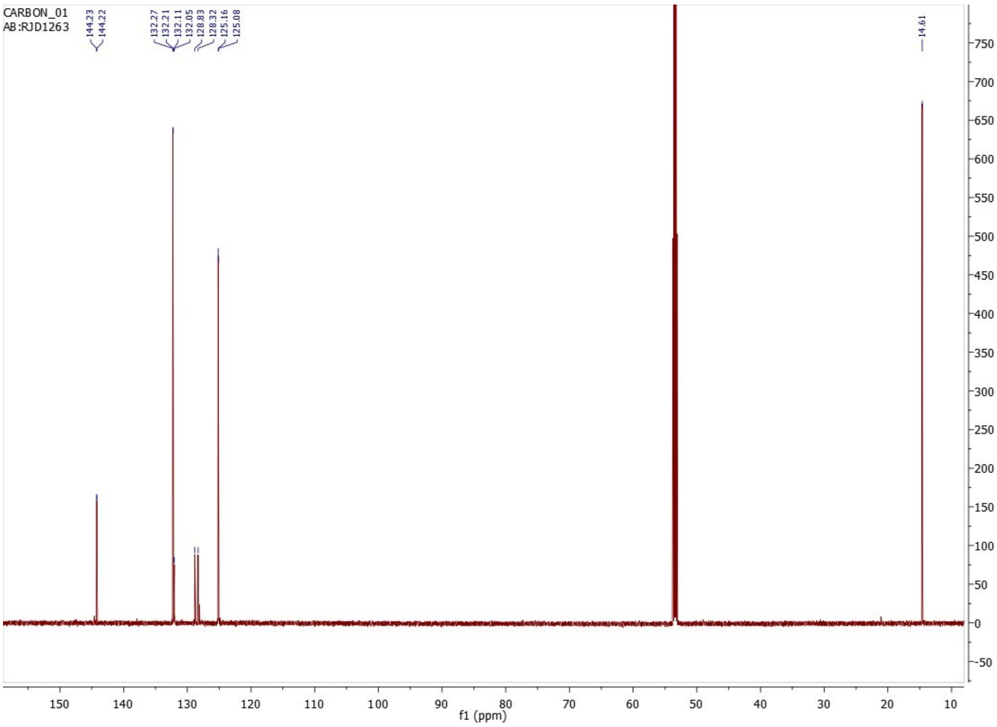

Figure S14. ^{13}C NMR of **3=S** recorded in CD_2Cl_2.

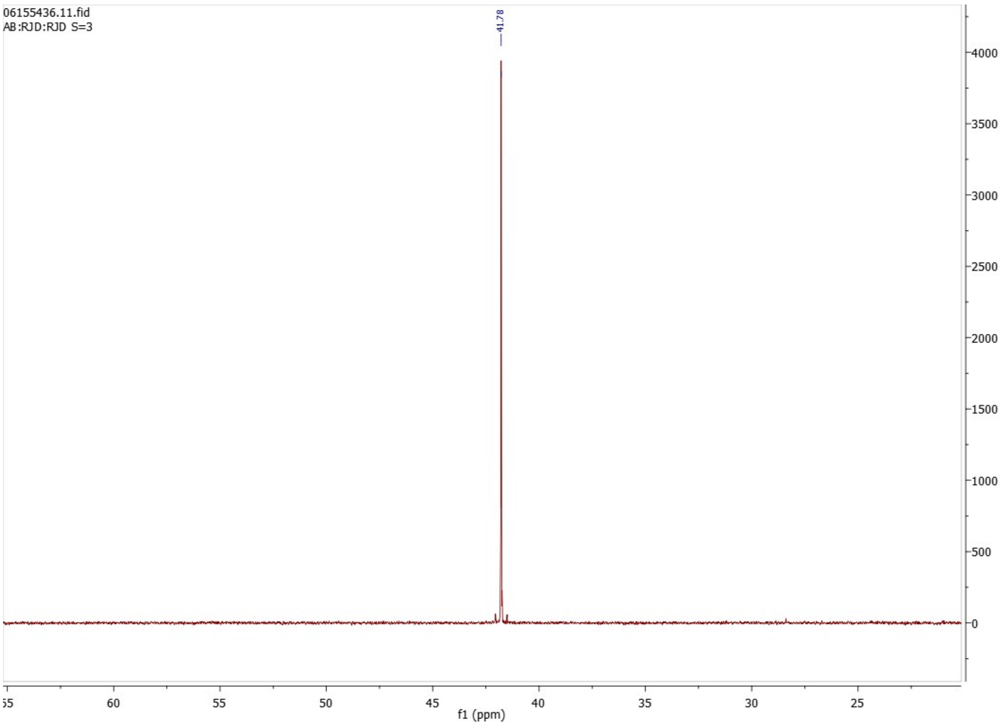

Figure S15. ^{31}P NMR of **3=S** recorded in CD_2Cl_2.

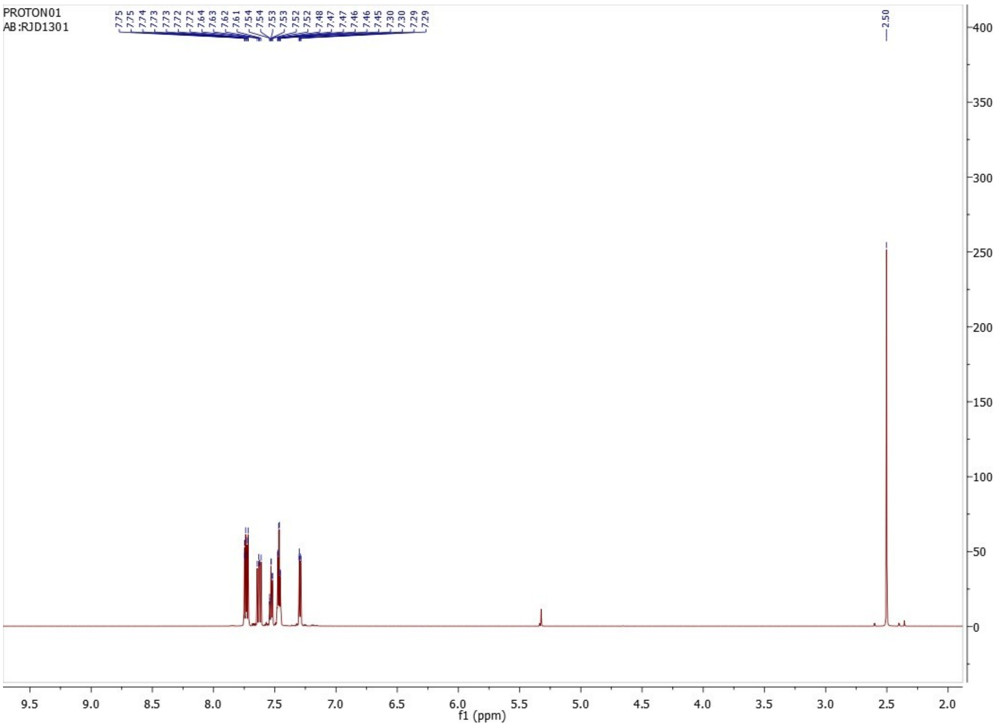

Figure S16. ^{1}H NMR of **1=Se** recorded in CD_2Cl_2.

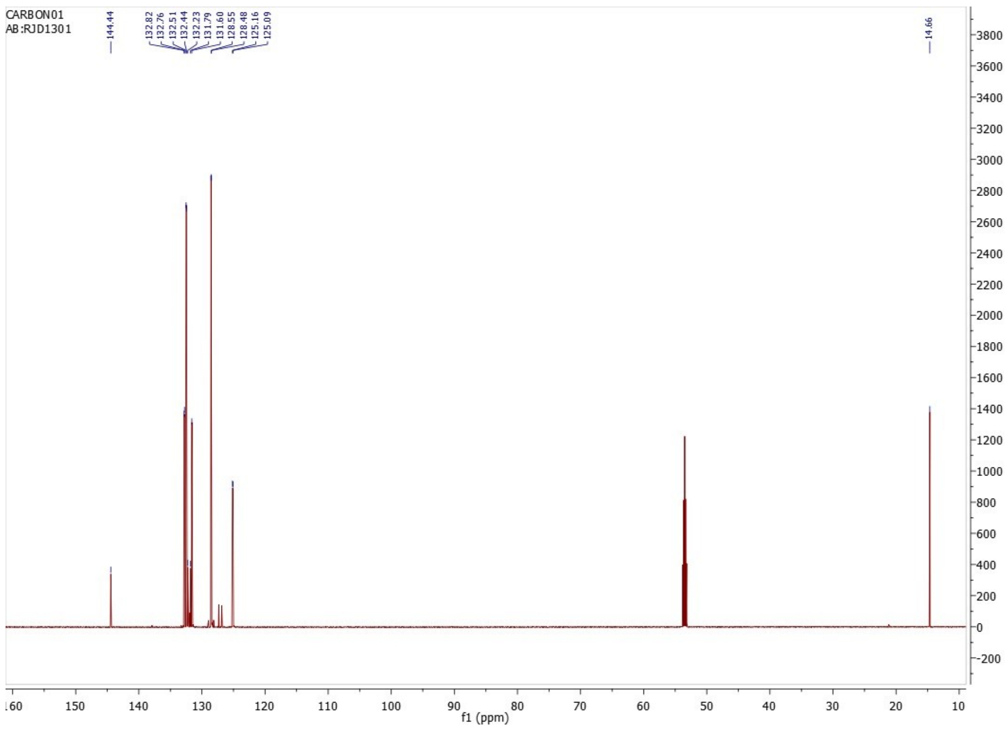

Figure S17. ^{13}C NMR of **1=Se** recorded in CD_2Cl_2.

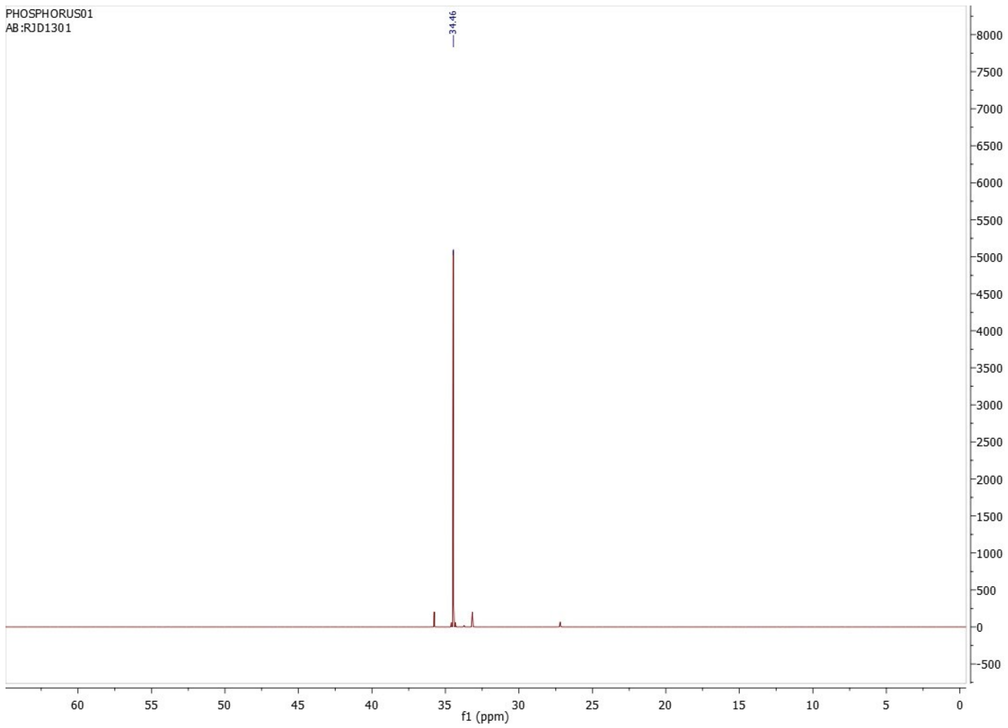

Figure S18. ^{31}P NMR of **1=Se** recorded in CD_2Cl_2.

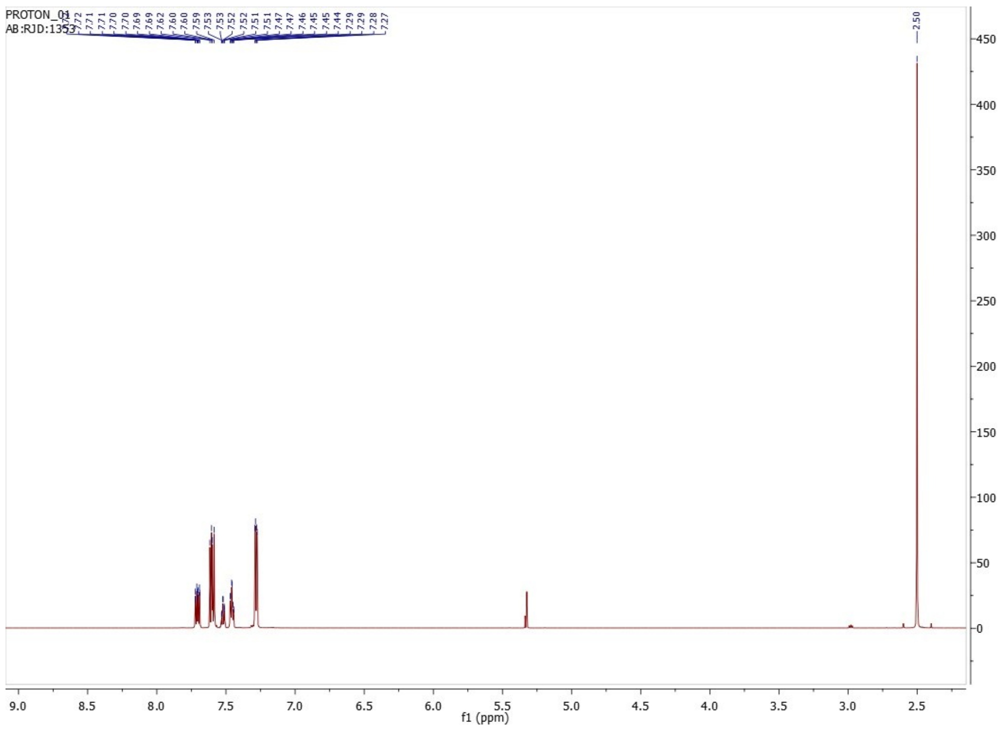

Figure S19. ^{1}H NMR of **2=Se** recorded in CD_2Cl_2.

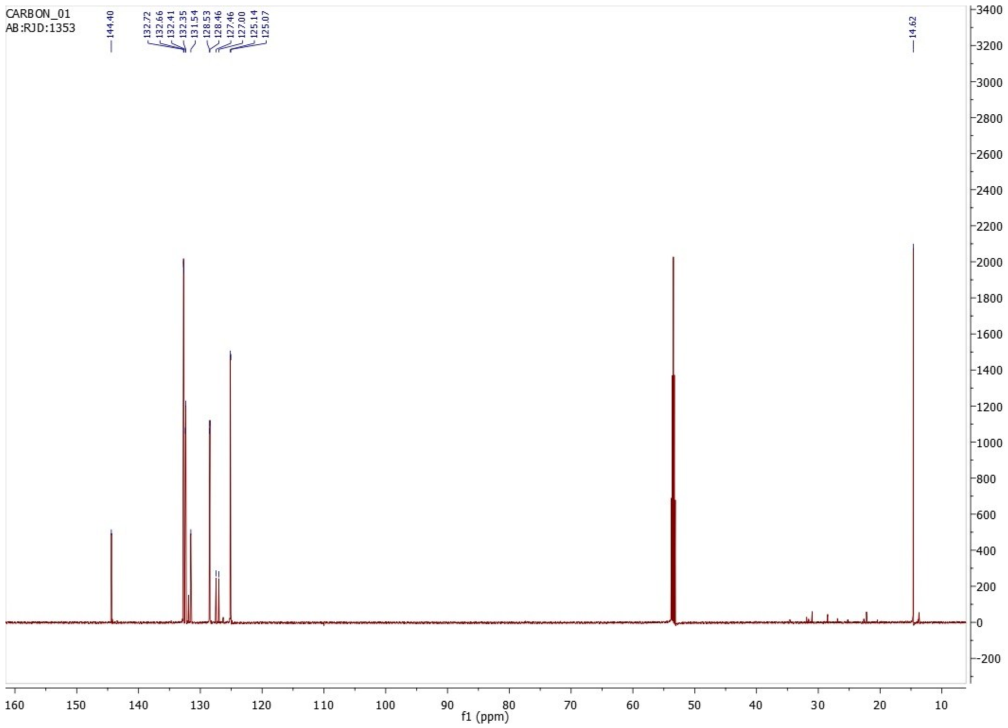

Figure S20. ^{13}C NMR of **2=Se** recorded in CD_2Cl_2.

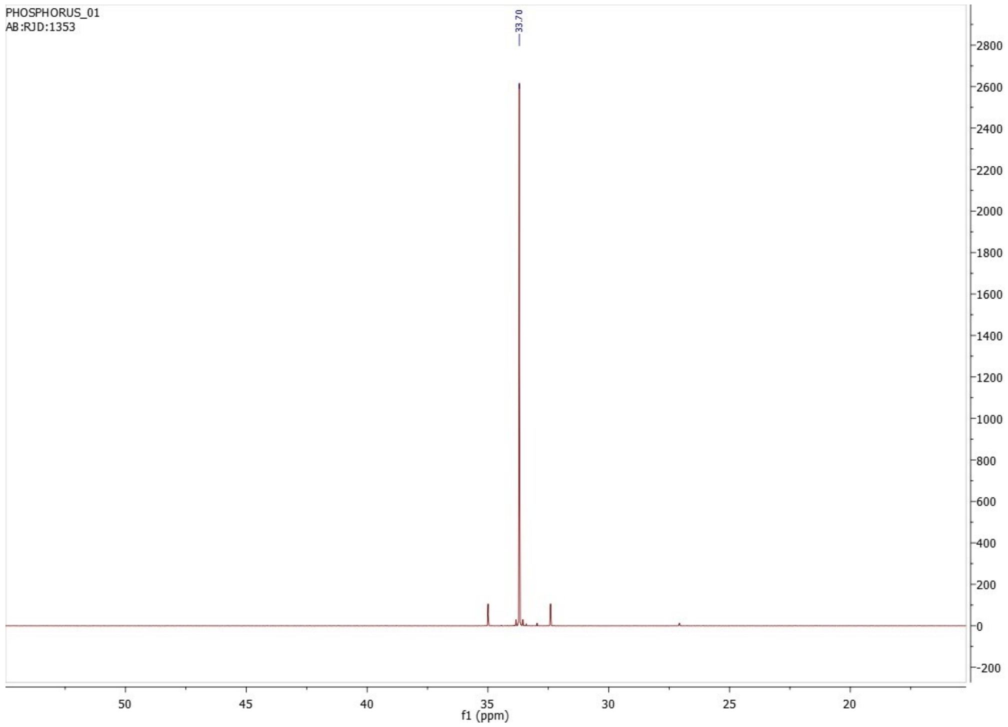

Figure S21. ^{31}P NMR of **2=Se** recorded in CD_2Cl_2.

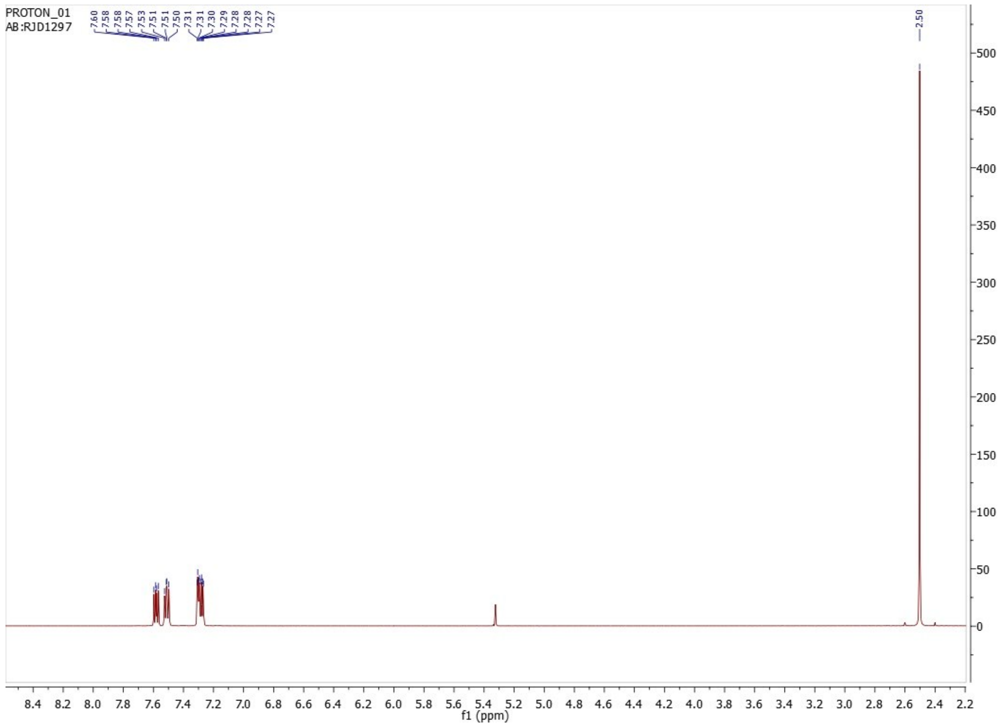

Figure S22. ^{1}H NMR of **3=Se** recorded in CD_2Cl_2.

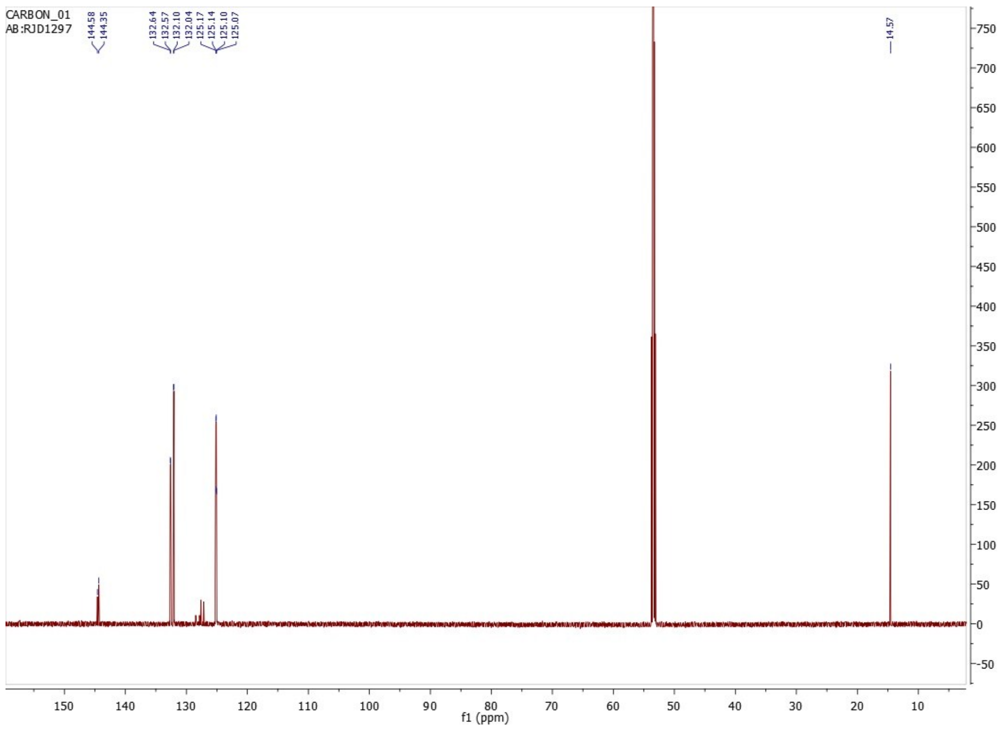

Figure S23. ^{13}C NMR of **3=Se** recorded in CD_2Cl_2.

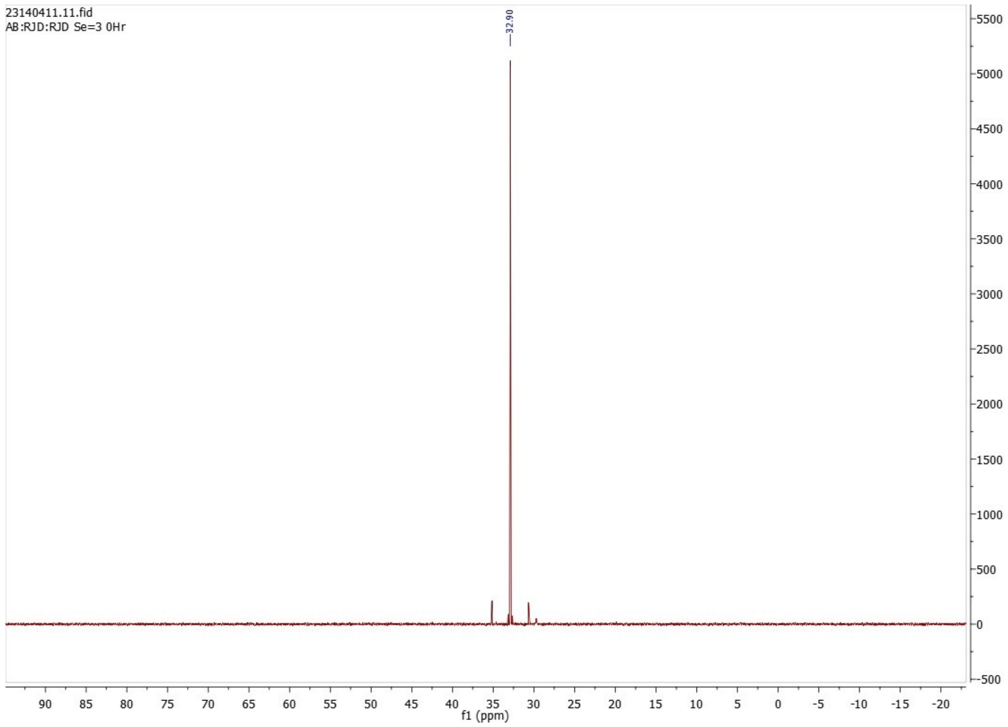

Figure S24. ^{31}P NMR of **3=Se** recorded in CD_2Cl_2.

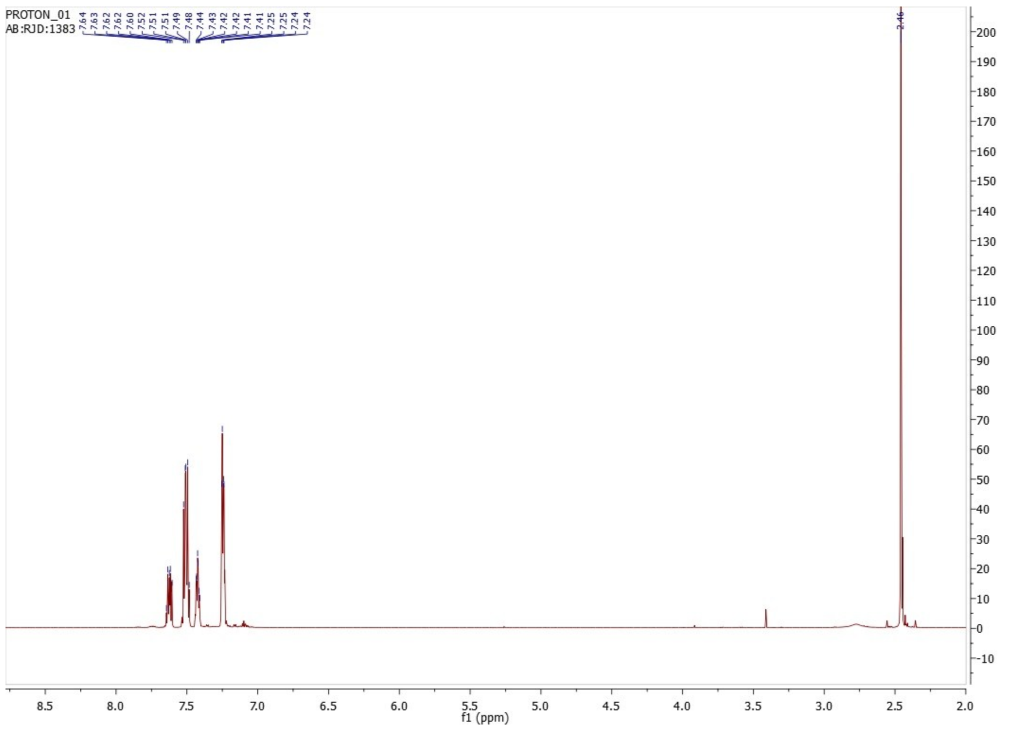

Figure S25. ^{1}H NMR of **2=O** recorded in $CDCl_3$.

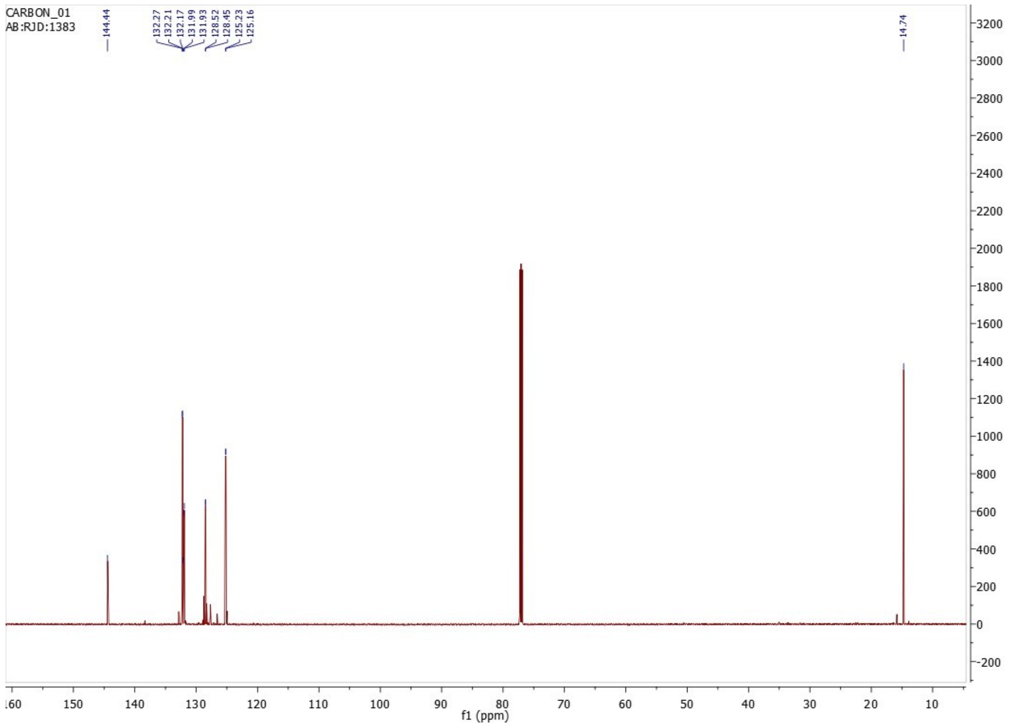

Figure S26. ^{13}C NMR of **2=O** recorded in $CDCl_3$.

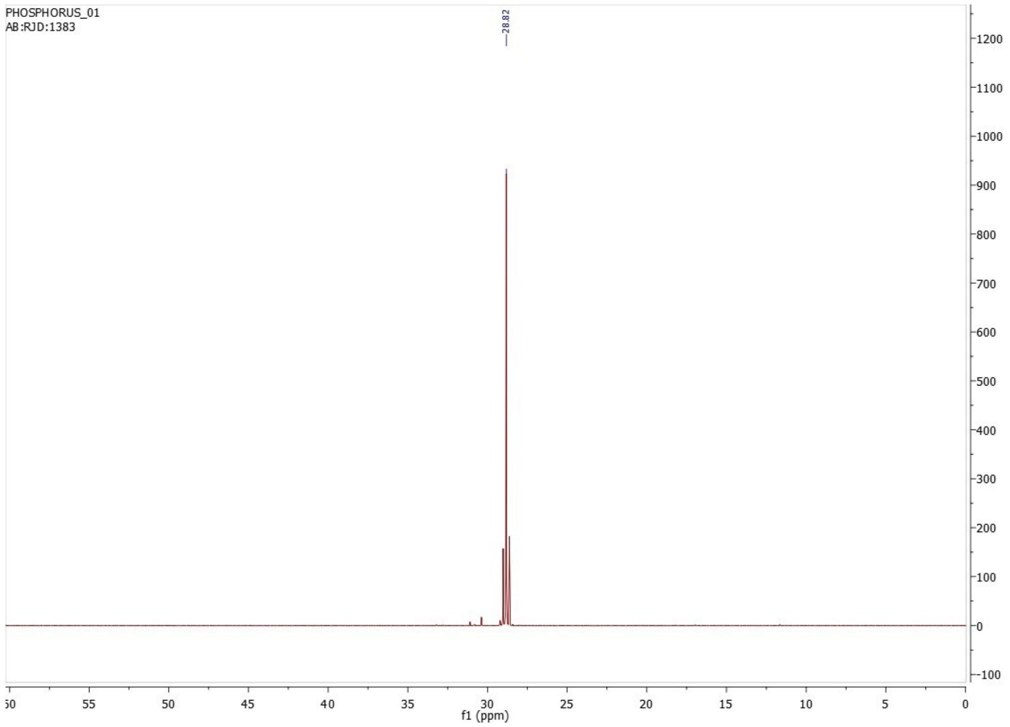

Figure S27. ^{31}P NMR of **2=O** recorded in $CDCl_3$.

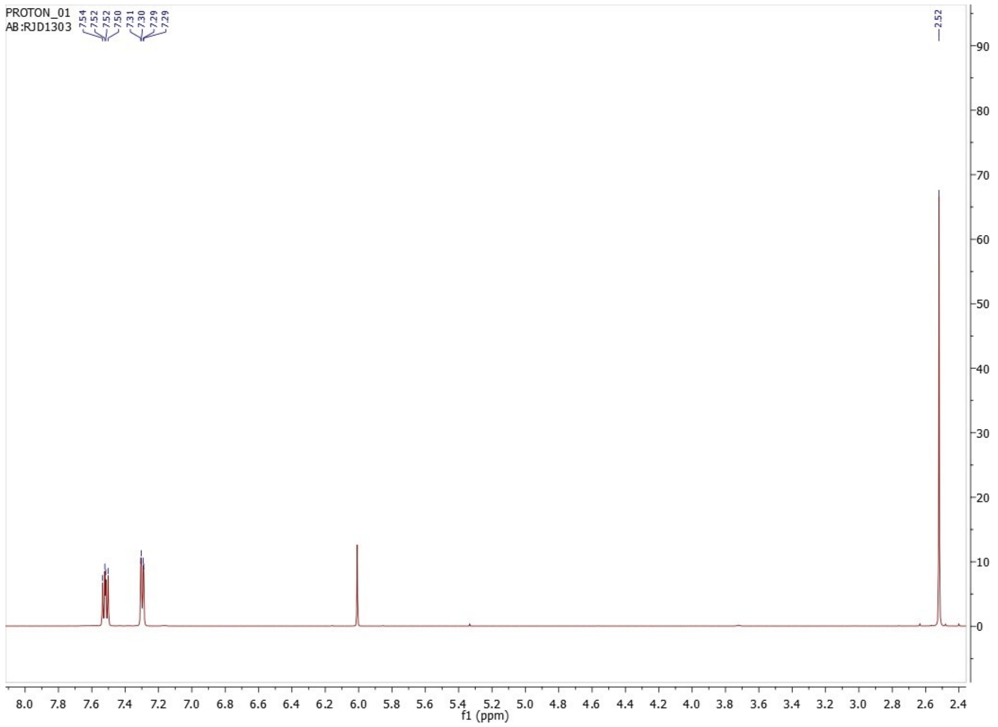

Figure S28. ^{1}H NMR of **3=O** recorded in TCE-d_2.

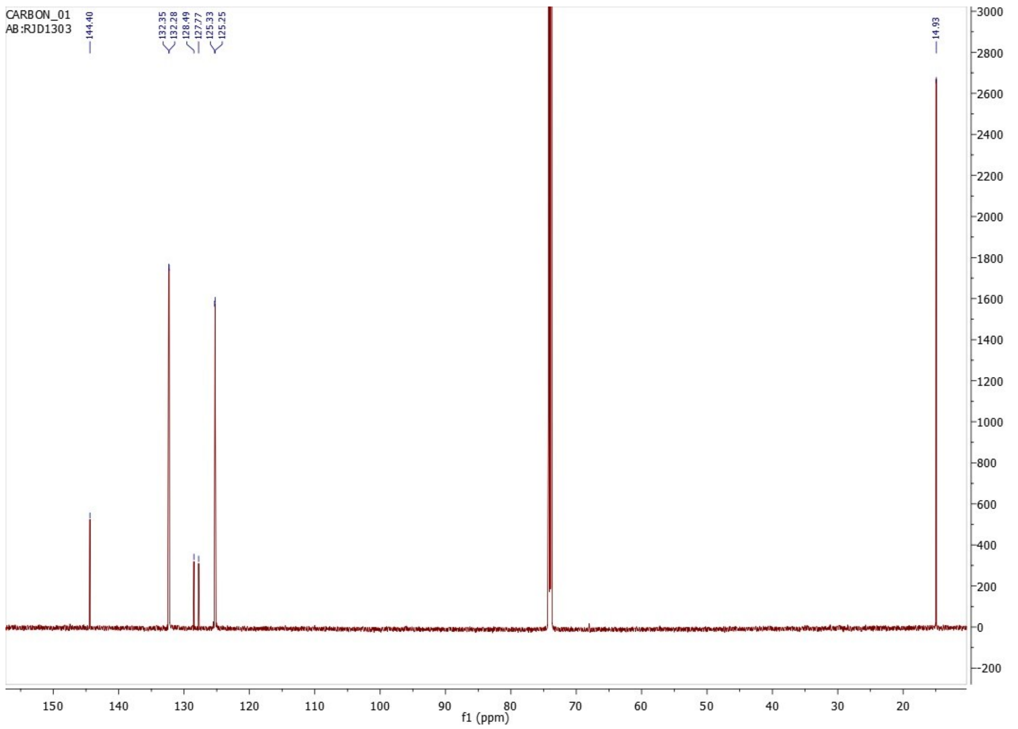

Figure S29. ^{13}C NMR of **3=O** recorded in TCE-d_2.

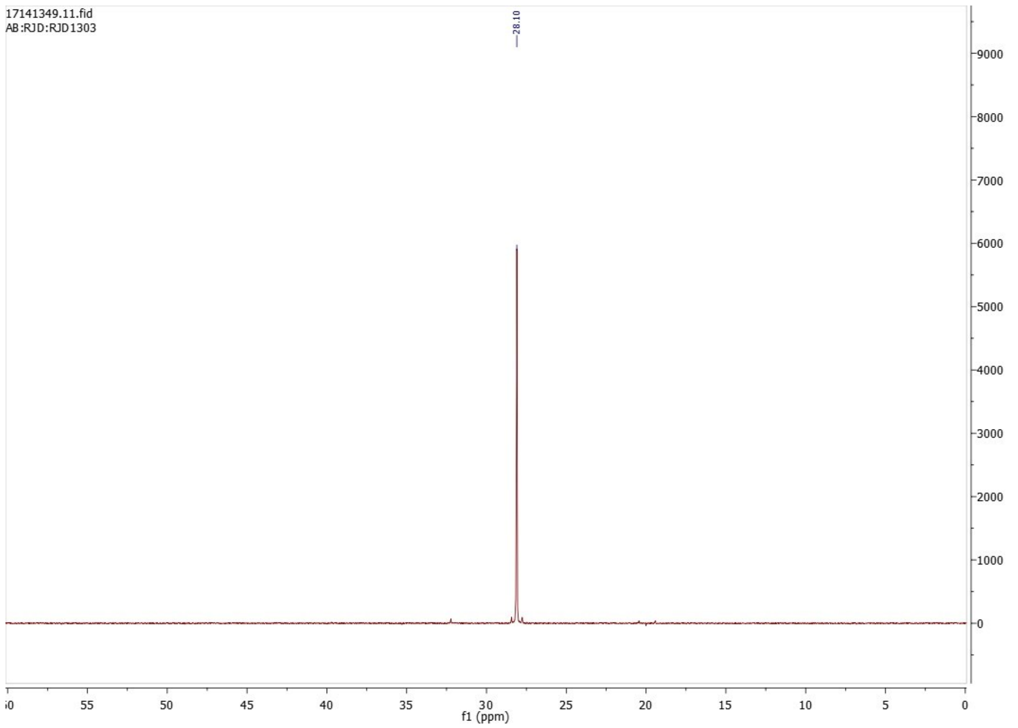

Figure S30. ^{31}P NMR of **3=O** recorded in TCE-d_2.

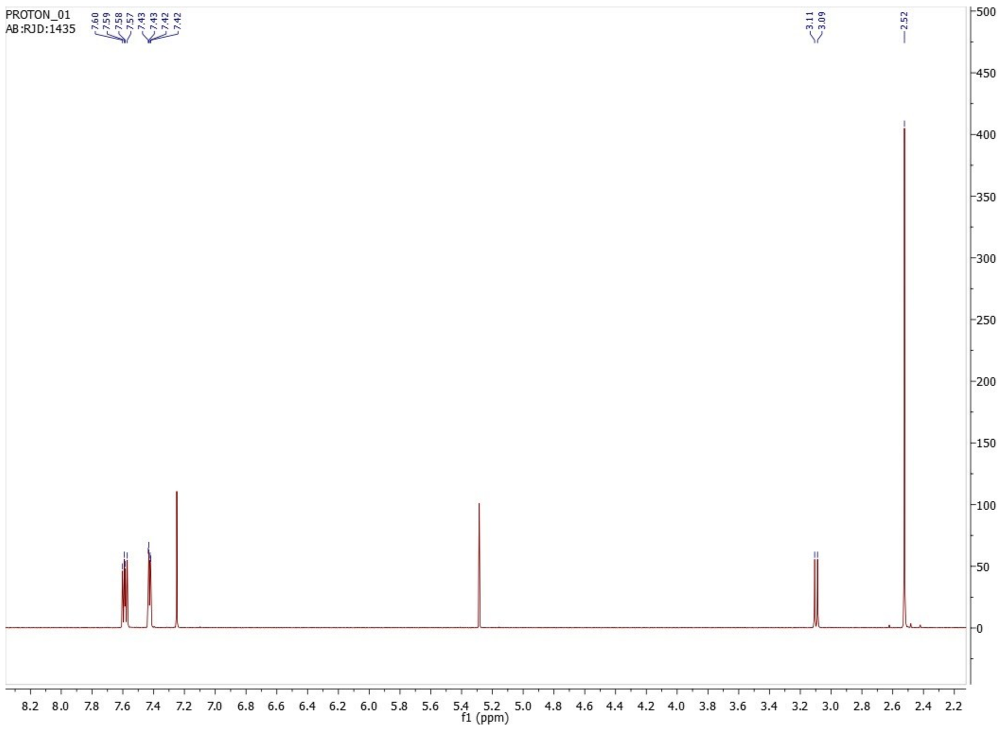

Figure S31. ^{1}H NMR of **[3-Me]**$^{+}$ recorded in $CDCl_3$.

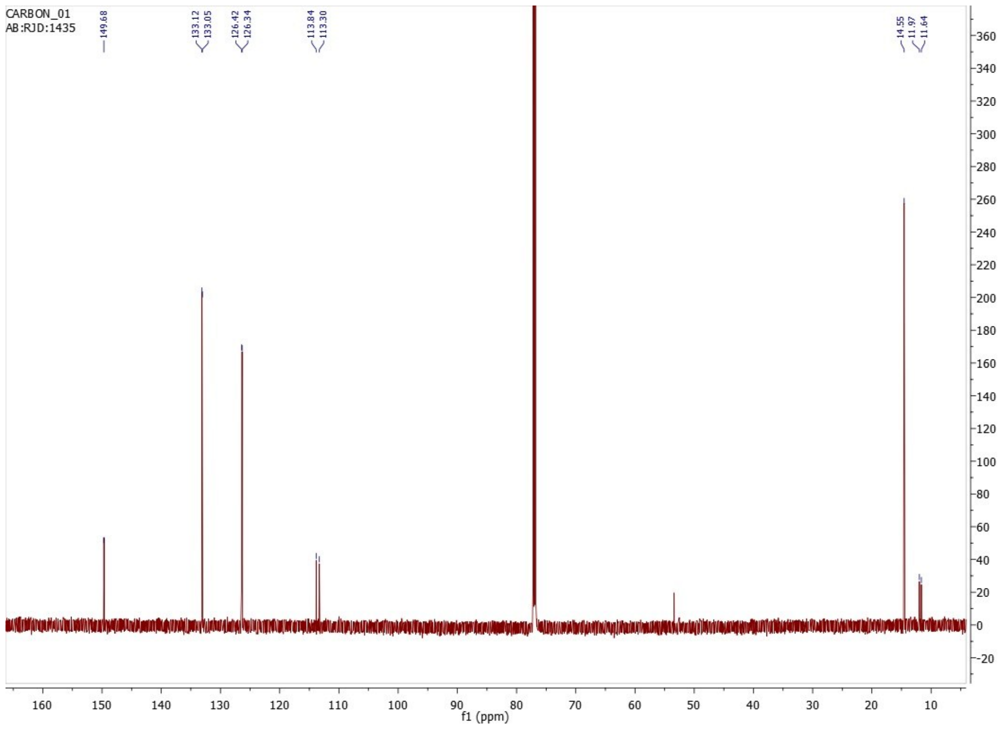

Figure S32. ^{13}C NMR of **[3-Me]**$^{+}$ recorded in $CDCl_3$.

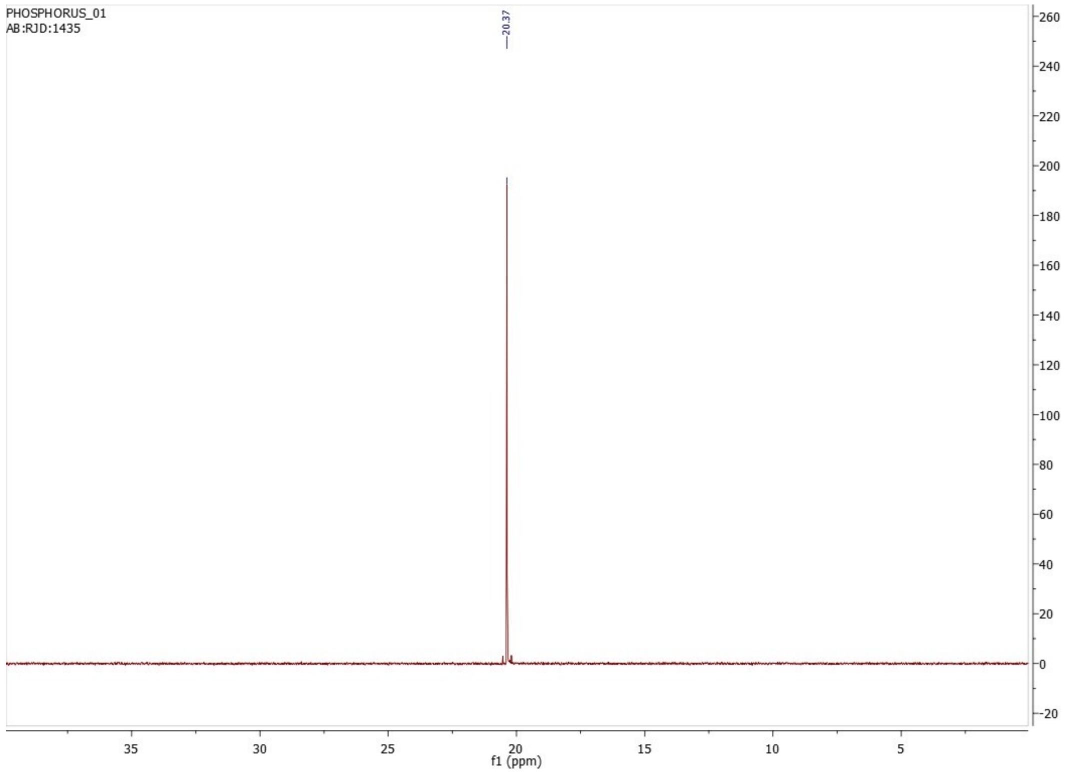

Figure S33. ^{31}P NMR of **[3-Me]$^+$** recorded in $CDCl_3$.

3. Phosphine selenide decay

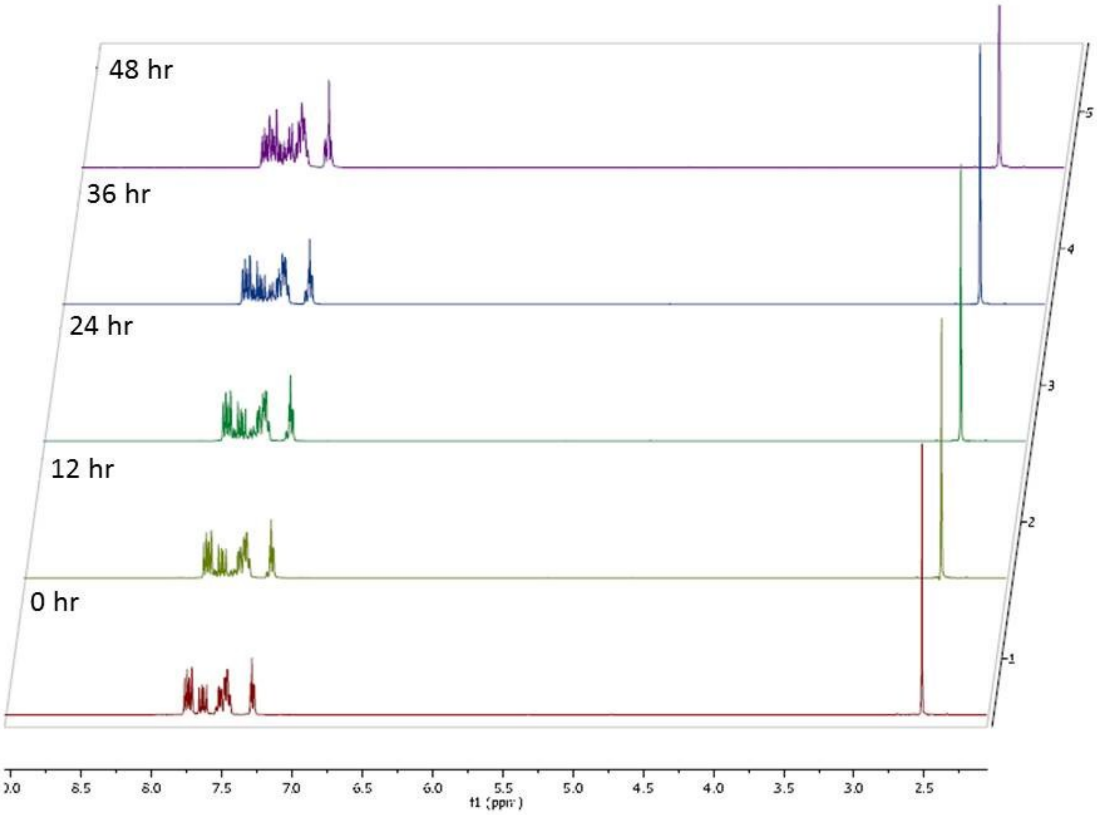

Figure S34. ^{1}H NMR spectra of **Se=2** over a period of 48 hours, recorded in $CDCl_3$.

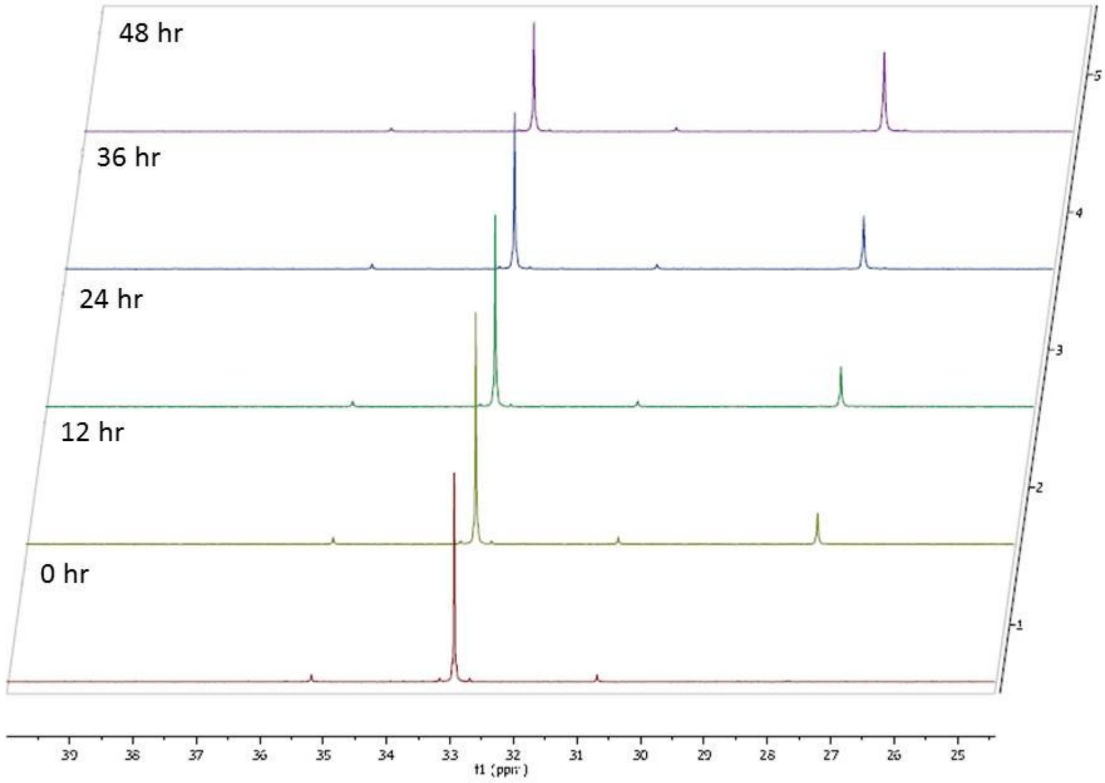

Figure S35. ^{31}P NMR spectra of **Se=2** over a period of 48 hours, recorded in $CDCl_3$.

4. Crystallographic data

The X-ray crystal structures of all **3=S**, **3=Se** and **3=O** show the typical pseudo-tetrahedral geometry of phosphorus atoms (Figure S36) with propeller-like orientation of aromatic rings and S-Me groups co-planar to corresponding Ph-rings. Predictably, the P=Q bond length (Q = O, S or Se) increases down the period: P=Se (2.1022 (4) Å) > P=S (1.9511 (5) Å) > P=O (1.4925 (12) Å). The 3=S and 3=Se compounds are isostructural and their packing motifs are determined by P=Q…S, C(Ph)H…Q and S…S intermolecular interactions. The unit cell of 3=O is similar to those of two other compounds but the space group and packing of the molecules is quite different. Only one direction-specific C(Ar)-H…O=P contact can be seen in structure **3=O**. No π…π interactions are present in these structures.

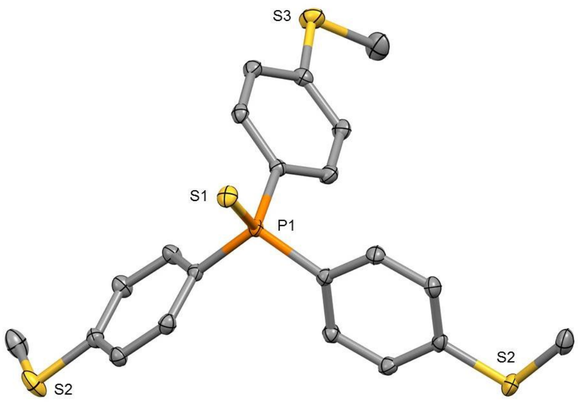

Figure S36. X-ray crystal structure of **3=S**, hydrogen atoms removed for clarity.

Table S1. Crystal and Refinement Data for 3=S, 3=Se, and 3=O.

**Compound**	**3=S**	**3=Se**	**3=O**
Code	15srv216	15srv218	16srv361
Empirical formula	$C_{21}H_{21}PS_4$	$C_{21}H_{21}PS_3Se$	$C_{21}H_{21}OPS_3$
Formula weight	432.59	479.49	416.53
Temperature/K	120.0	120.0	120.0
Crystal system	monoclinic	monoclinic	monoclinic
Space group	$P2_1/c$	$P2_1/c$	$P2_1/n$
a/Å	10.7372(7)	10.8302(4)	10.1402(5)
b/Å	19.6819(13)	19.7686(8)	20.3778(10)
c/Å	10.9938(7)	10.9470(4)	10.7202(5)
α/°	90.00	90.00	90
β/°	114.163(2)	112.8736(13)	114.9689(16)
γ/°	90.00	90.00	90
Volume/$Å^3$	2119.7(2)	2159.43(14)	2008.13(17)
Z	4	4	4
$\rho_{calc}g/cm^3$	1.356	1.475	1.378
μ/mm^{-1}	0.527	2.106	0.457
F(000)	904.0	976.0	872.0
Crystal size/mm^3	0.35 × 0.16 × 0.14	0.48 × 0.24 × 0.24	0.15 × 0.11 × 0.07
Radiation	MoKα (λ = 0.71073)	MoKα (λ = 0.71073)	MoKα (λ = 0.71073)
2Θ range for data collection/°	4.14 to 58	4.54 to 58	4.64 to 59.998
Index ranges	-14 ≤ h ≤ 14, -26 ≤ k ≤ 26, -14 ≤ l ≤ 14	-14 ≤ h ≤ 14, -26 ≤ k ≤ 26, -14 ≤ l ≤ 14	-14 ≤ h ≤ 14, -28 ≤ k ≤ 28, -15 ≤ l ≤ 15
Reflections collected	32442	33209	44040
Independent reflections	5625 [R_{int} = 0.0390, R_{sigma} = 0.0298]	5737 [R_{int} = 0.0293, R_{sigma} = 0.0210]	5855 [R_{int} = 0.0447, R_{sigma} = 0.0301]
Data/restraints/parameters	5625/0/319	5737/0/319	5855/0/319
Goodness-of-fit on F^2	1.055	1.074	1.038
Final R indexes [I>=2σ (I)]	R_1 = 0.0337, wR_2 = 0.0809	R_1 = 0.0260, wR_2 = 0.0608	R_1 = 0.0344, wR_2 = 0.0755
Final R indexes [all data]	R_1 = 0.0431, wR_2 = 0.0853	R_1 = 0.0314, wR_2 = 0.0631	R_1 = 0.0483, wR_2 = 0.0807
Largest diff. peak/hole / e $Å^{-3}$	0.45/-0.34	0.94/-0.43	0.51/-0.25

Table S2. Selected bond lengths for **3=S**.

Atom	Atom	Length/Å		Atom	Atom	Length/Å
S1	P1	1.9511(5)		C4	C5	1.397(2)
S2	C4	1.7563(15)		C5	C6	1.389(2)
S2	C7	1.7952(19)		C8	C9	1.391(2)
S3	C11	1.7520(15)		C8	C13	1.401(2)
S3	C14	1.7901(19)		C9	C10	1.392(2)
S4	C18	1.7518(15)		C10	C11	1.393(2)
S4	C21	1.7939(19)		C11	C12	1.402(2)
P1	C1	1.8033(15)		C12	C13	1.378(2)
P1	C8	1.8054(15)		C15	C16	1.392(2)
P1	C15	1.8072(15)		C15	C20	1.403(2)
C1	C2	1.396(2)		C16	C17	1.389(2)
C1	C6	1.395(2)		C17	C18	1.390(2)
C2	C3	1.387(2)		C18	C19	1.406(2)
C3	C4	1.394(2)		C19	C20	1.381(2)

Table S3. Selected bond angles for **3=S**.

Atom	Atom	Atom	Angle/°		Atom	Atom	Atom	Angle/°
C4	S2	C7	103.89(8)		C9	C8	C13	118.49(14)
C11	S3	C14	103.41(8)		C13	C8	P1	119.37(11)
C18	S4	C21	102.96(8)		C8	C9	C10	121.16(14)
C1	P1	S1	113.17(5)		C9	C10	C11	119.99(14)
C1	P1	C8	103.82(7)		C10	C11	S3	124.84(12)
C1	P1	C15	107.76(7)		C10	C11	C12	118.96(14)
C8	P1	S1	114.52(5)		C12	C11	S3	116.21(11)
C8	P1	C15	104.46(6)		C13	C12	C11	120.72(14)
C15	P1	S1	112.33(5)		C12	C13	C8	120.64(14)
C2	C1	P1	122.91(11)		C16	C15	P1	121.66(11)
C6	C1	P1	117.98(11)		C16	C15	C20	118.78(13)
C6	C1	C2	118.95(13)		C20	C15	P1	119.38(11)
C3	C2	C1	120.22(14)		C17	C16	C15	120.96(14)
C2	C3	C4	120.64(14)		C16	C17	C18	120.30(14)
C3	C4	S2	116.34(11)		C17	C18	S4	124.49(12)
C3	C4	C5	119.38(13)		C17	C18	C19	118.95(14)
C5	C4	S2	124.26(12)		C19	C18	S4	116.55(11)
C6	C5	C4	119.71(14)		C20	C19	C18	120.59(14)
C5	C6	C1	121.01(14)		C19	C20	C15	120.40(14)
C9	C8	P1	122.01(11)					

Table S4. Selected bond lengths for **3=Se**.

Atom	Atom	Length/Å		Atom	Atom	Length/Å
Se1	P1	2.1022(4)		C4	C5	1.396(2)
S1	C4	1.7533(16)		C5	C6	1.392(2)
S1	C7	1.791(2)		C8	C9	1.396(2)
S2	C11	1.7538(15)		C8	C13	1.392(2)
S2	C14	1.797(2)		C9	C10	1.386(2)
S3	C18	1.7520(16)		C10	C11	1.396(2)
S3	C21	1.794(2)		C11	C12	1.400(2)
P1	C1	1.8082(16)		C12	C13	1.385(2)
P1	C8	1.8076(15)		C15	C16	1.391(2)
P1	C15	1.8054(15)		C15	C20	1.403(2)
C1	C2	1.397(2)		C16	C17	1.391(2)
C1	C6	1.392(2)		C17	C18	1.390(2)
C2	C3	1.379(2)		C18	C19	1.405(2)
C3	C4	1.398(2)		C19	C20	1.381(2)

Table S5. Selected bond angles for **3=Se**.

Atom	Atom	Atom	Angle/°		Atom	Atom	Atom	Angle/°
C4	S1	C7	103.46(9)		C13	C8	P1	122.87(12)
C11	S2	C14	103.99(9)		C13	C8	C9	119.18(14)
C18	S3	C21	103.05(9)		C10	C9	C8	120.75(16)
C1	P1	Se1	114.66(5)		C9	C10	C11	119.96(15)
C8	P1	Se1	112.62(5)		C10	C11	S2	124.21(12)
C8	P1	C1	104.09(7)		C10	C11	C12	119.27(14)
C15	P1	Se1	111.97(5)		C12	C11	S2	116.50(12)
C15	P1	C1	104.72(7)		C13	C12	C11	120.42(15)
C15	P1	C8	108.12(7)		C12	C13	C8	120.36(15)
C2	C1	P1	119.65(12)		C16	C15	P1	121.53(12)
C6	C1	P1	121.45(12)		C16	C15	C20	118.97(14)
C6	C1	C2	118.76(14)		C20	C15	P1	119.31(11)
C3	C2	C1	120.52(15)		C17	C16	C15	120.82(15)
C2	C3	C4	120.79(15)		C18	C17	C16	120.24(15)
C3	C4	S1	116.23(12)		C17	C18	S3	124.51(12)
C5	C4	S1	124.81(13)		C17	C18	C19	119.07(14)
C5	C4	C3	118.97(15)		C19	C18	S3	116.41(12)
C6	C5	C4	119.92(15)		C20	C19	C18	120.61(15)
C1	C6	C5	120.97(15)		C19	C20	C15	120.28(14)
C9	C8	P1	117.85(12)					

Table S6. Selected bond lengths for **3=O**.

Atom	Atom	Length/Å		Atom	Atom	Length/Å
S1	C4	1.7581(14)		C4	C5	1.396(2)
S1	C7	1.7982(17)		C5	C6	1.390(2)
S2	C11	1.7572(14)		C8	C9	1.3937(19)
S2	C14	1.7972(18)		C8	C13	1.4005(19)
S3	C18	1.7576(14)		C9	C10	1.3923(19)
S3	C21	1.7953(18)		C10	C11	1.3937(19)
P1	O1	1.4925(10)		C11	C12	1.397(2)
P1	C1	1.8003(14)		C12	C13	1.381(2)
P1	C8	1.7979(14)		C15	C16	1.397(2)
P1	C15	1.8002(14)		C15	C20	1.3938(19)
C1	C2	1.3979(19)		C16	C17	1.386(2)
C1	C6	1.395(2)		C17	C18	1.400(2)
C2	C3	1.383(2)		C18	C19	1.390(2)
C3	C4	1.397(2)		C19	C20	1.3922(19)

Table S7. Selected bond angles for **3=O**.

Atom	Atom	Atom	Angle/°		Atom	Atom	Atom	Angle/°
C4	S1	C7	103.63(7)		C9	C8	C13	118.72(13)
C11	S2	C14	104.14(7)		C13	C8	P1	117.90(10)
C18	S3	C21	103.12(7)		C10	C9	C8	120.98(13)
O1	P1	C1	112.04(6)		C9	C10	C11	119.69(13)
O1	P1	C8	111.66(6)		C10	C11	S2	124.00(11)
O1	P1	C15	110.57(6)		C10	C11	C12	119.63(13)
C8	P1	C1	107.33(6)		C12	C11	S2	116.37(11)
C8	P1	C15	108.01(6)		C13	C12	C11	120.34(13)
C15	P1	C1	107.00(6)		C12	C13	C8	120.62(13)
C2	C1	P1	121.64(11)		C16	C15	P1	123.83(11)
C6	C1	P1	118.98(11)		C20	C15	P1	117.30(10)
C6	C1	C2	118.68(13)		C20	C15	C16	118.86(13)
C3	C2	C1	120.37(13)		C17	C16	C15	120.25(13)
C2	C3	C4	120.79(13)		C16	C17	C18	120.71(13)
C3	C4	S1	116.81(11)		C17	C18	S3	116.48(11)
C5	C4	S1	124.03(11)		C19	C18	S3	124.28(11)
C5	C4	C3	119.15(13)		C19	C18	C17	119.24(13)
C6	C5	C4	119.79(13)		C18	C19	C20	119.88(13)
C5	C6	C1	121.13(13)		C19	C20	C15	121.06(13)
C9	C8	P1	123.33(11)					

b) Conductance Measurements

1. Conductance 2D Histograms

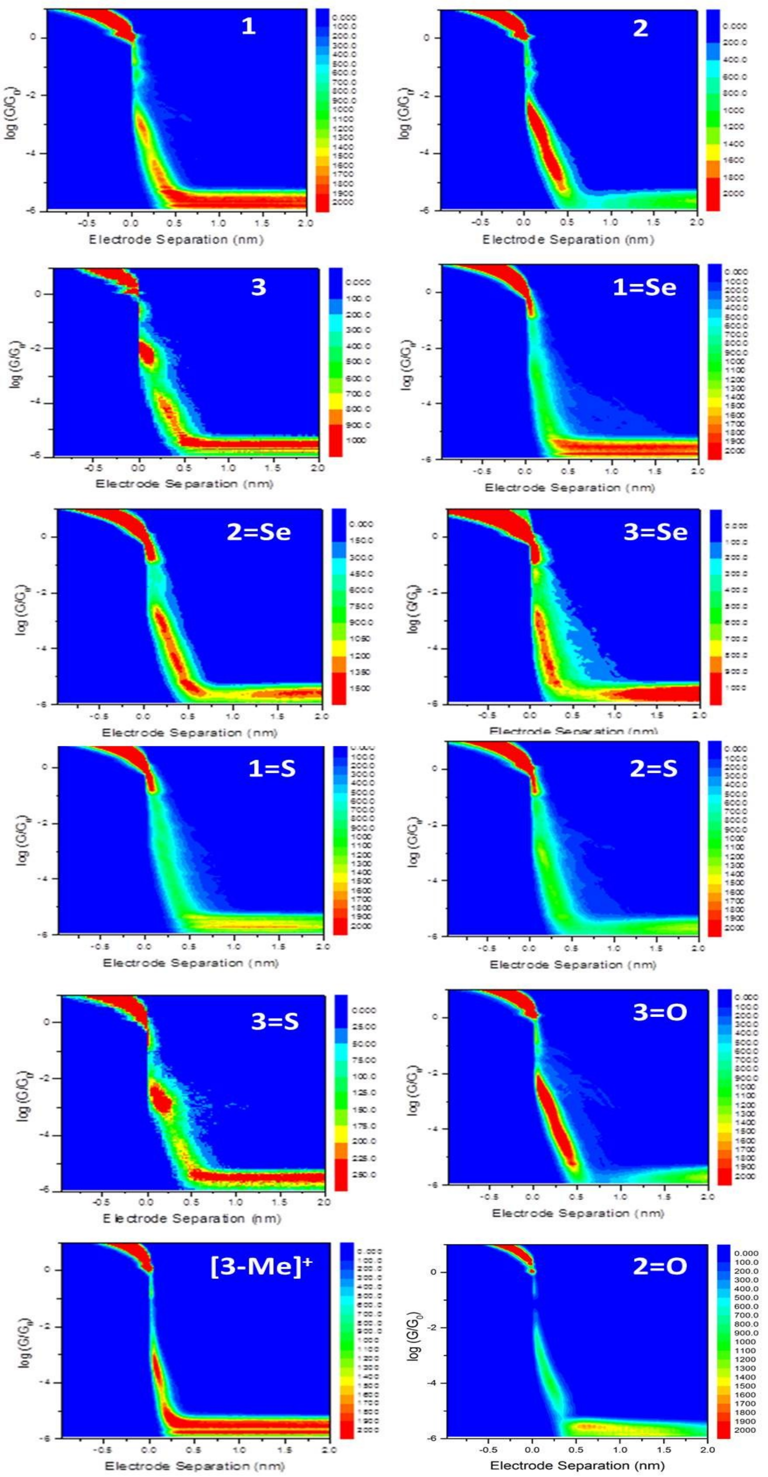

Figure S37. 2D conductance histograms for the compounds.

2. Conductance 1D Histograms

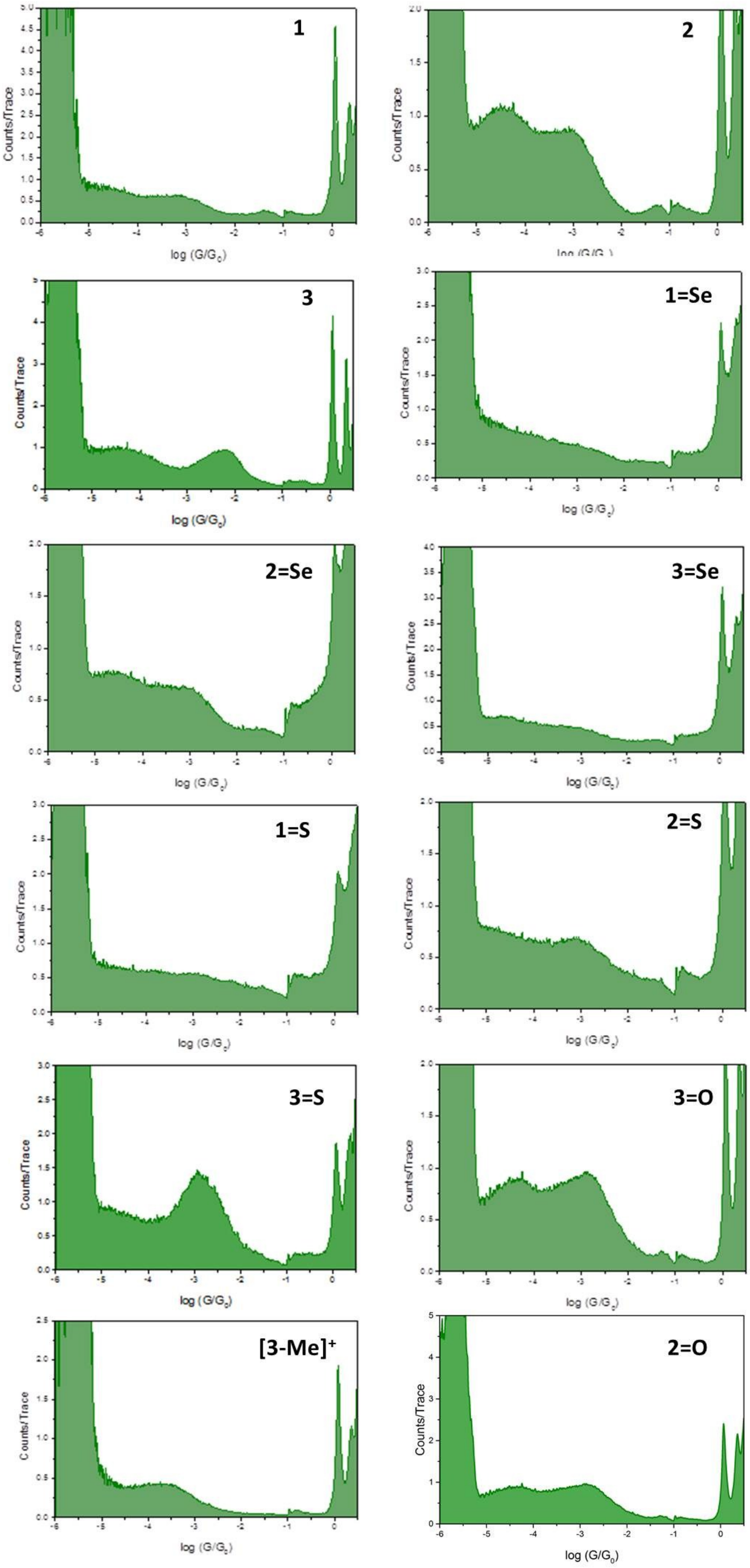

Figure S38. 1D conductance histograms for the compounds.

3. Representative conductance traces

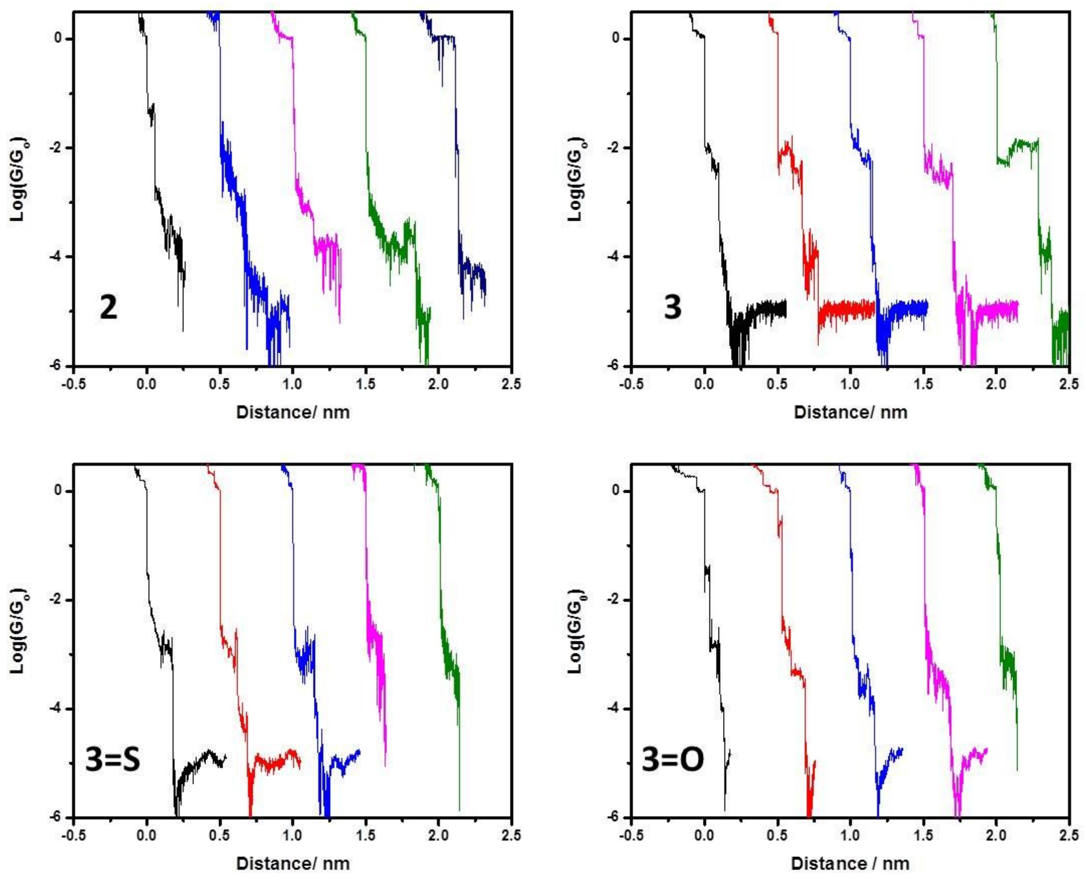

Figure S39. Five representative and arbitrarily chosen BJ curves for each molecules 2, 3, 3=S and 3=O, horizontally shifted.

c) Theoretical details

1. Geometry of isolated molecules

The DFT code (SIESTA) with Van der Waals exchange-correlation was used to obtain fully relaxed geometries of the isolated tripod molecules, as shown in Figure S40.

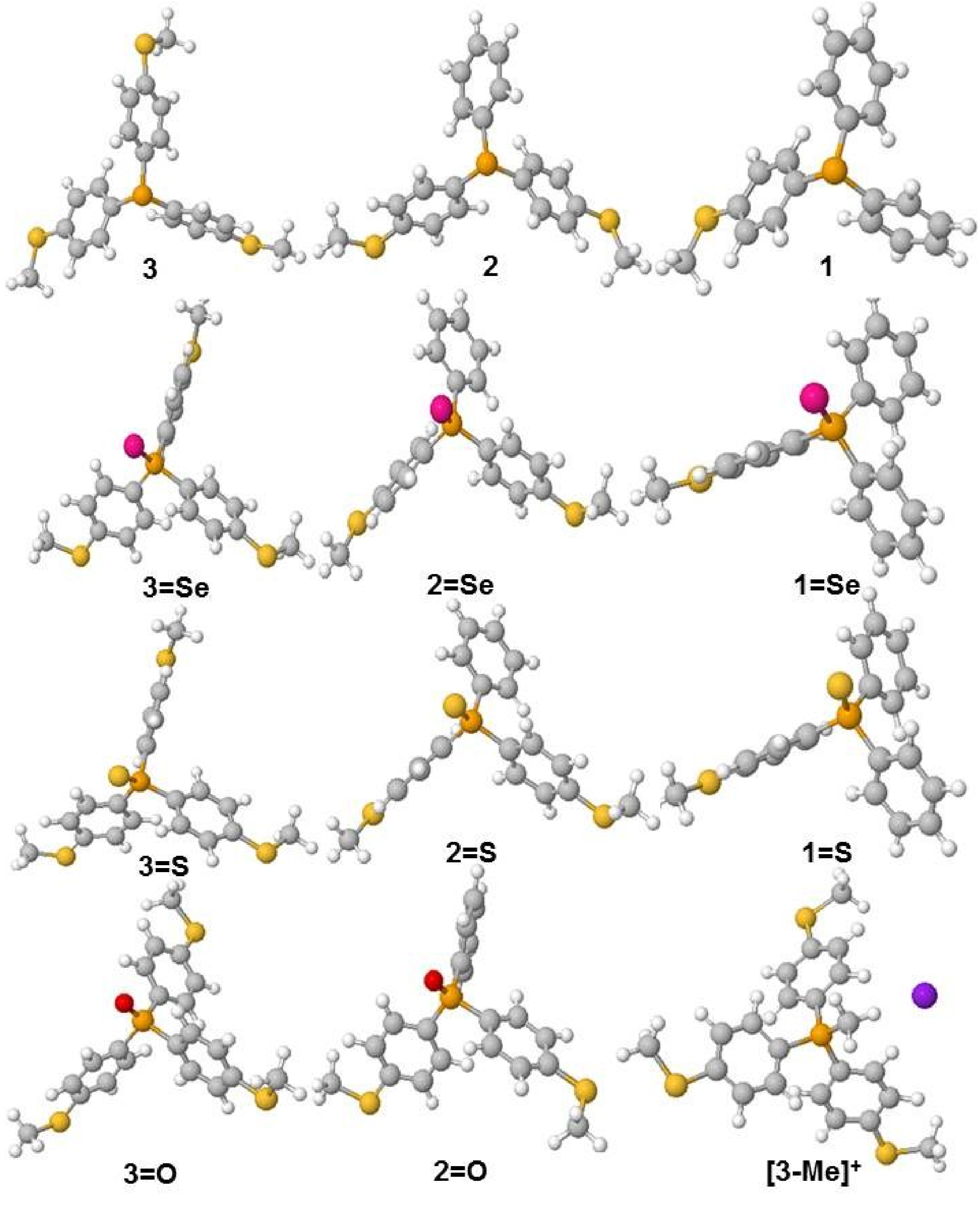

Figure S40. Fully relaxed isolated structures of the 12 tripod molecules in Chart 1 of the main text.

2. Binding energy of 2 (A, B, and C) and 3 on gold

We have calculated the binding for geometry locations about the 'relaxed' complex geometry when the **2-A** is situated above the gold electrode. We move the **2-A** in 3 dimensions at 252 different positions, along the x-direction it is moved 5Å from equilibrium, in the y-direction 10Å and in the z-direction 6Å. Figure S41 shows that there are many local minima around the global minimum. We repeat the same calculations for **2-B, 2-C** and **3** as shown in Figures S42, S43, and S44.

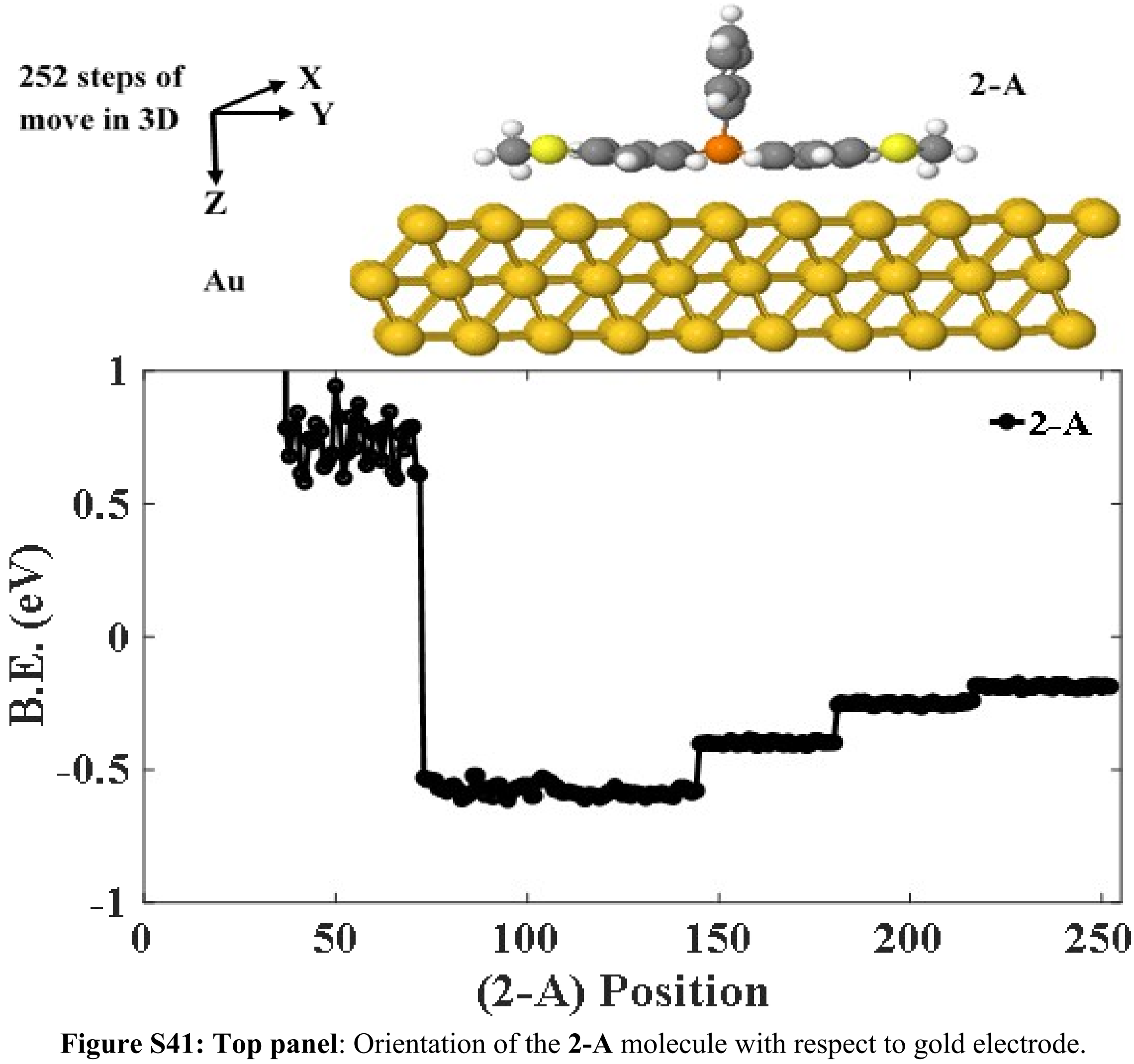

Figure S41: Top panel: Orientation of the **2-A** molecule with respect to gold electrode. **Lower panel**: Binding energy of **2-A** to gold electrode as a function of position.

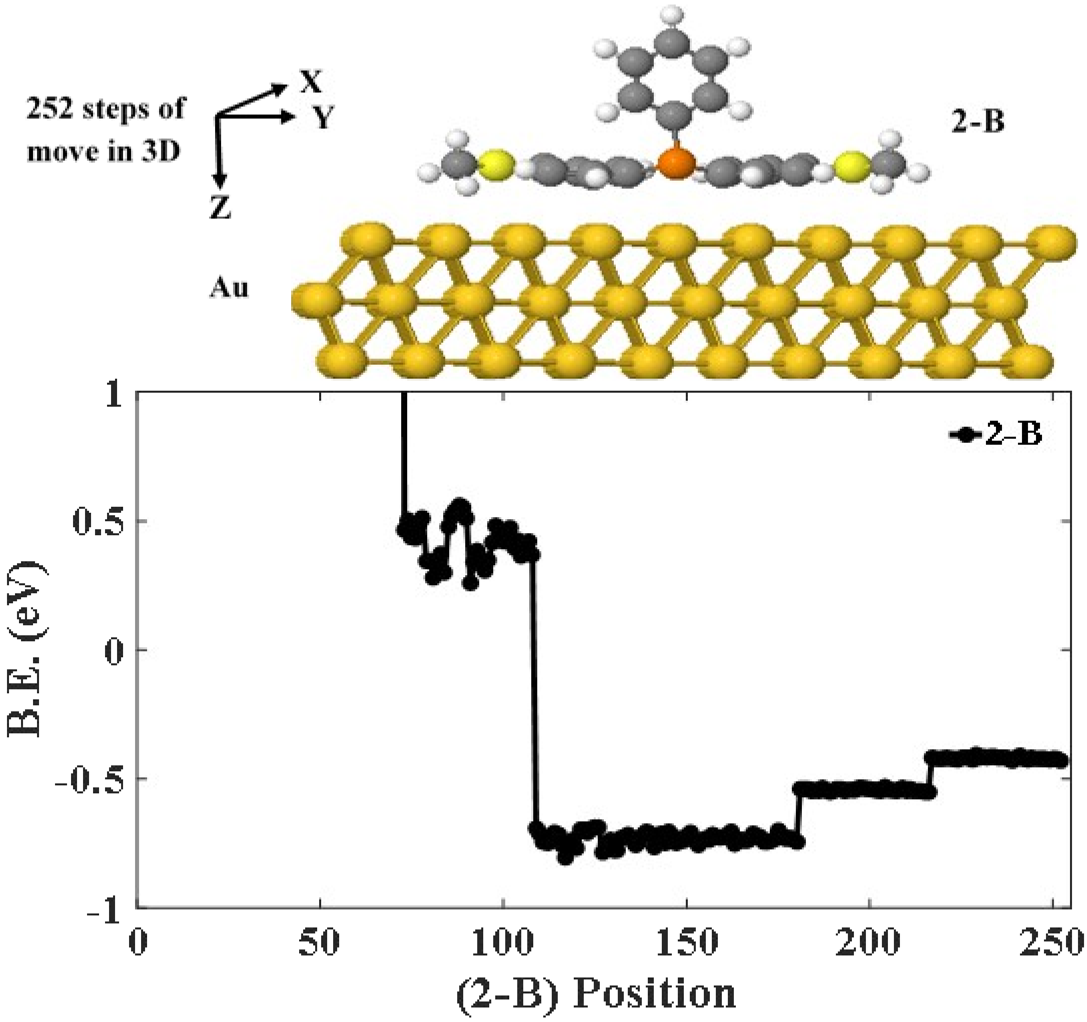

Figure S42: Top panel: Orientation of the **2-B** molecule with respect to gold electrode. **Lower panel**: Binding energy of **2-B** to gold electrode as a function of position.

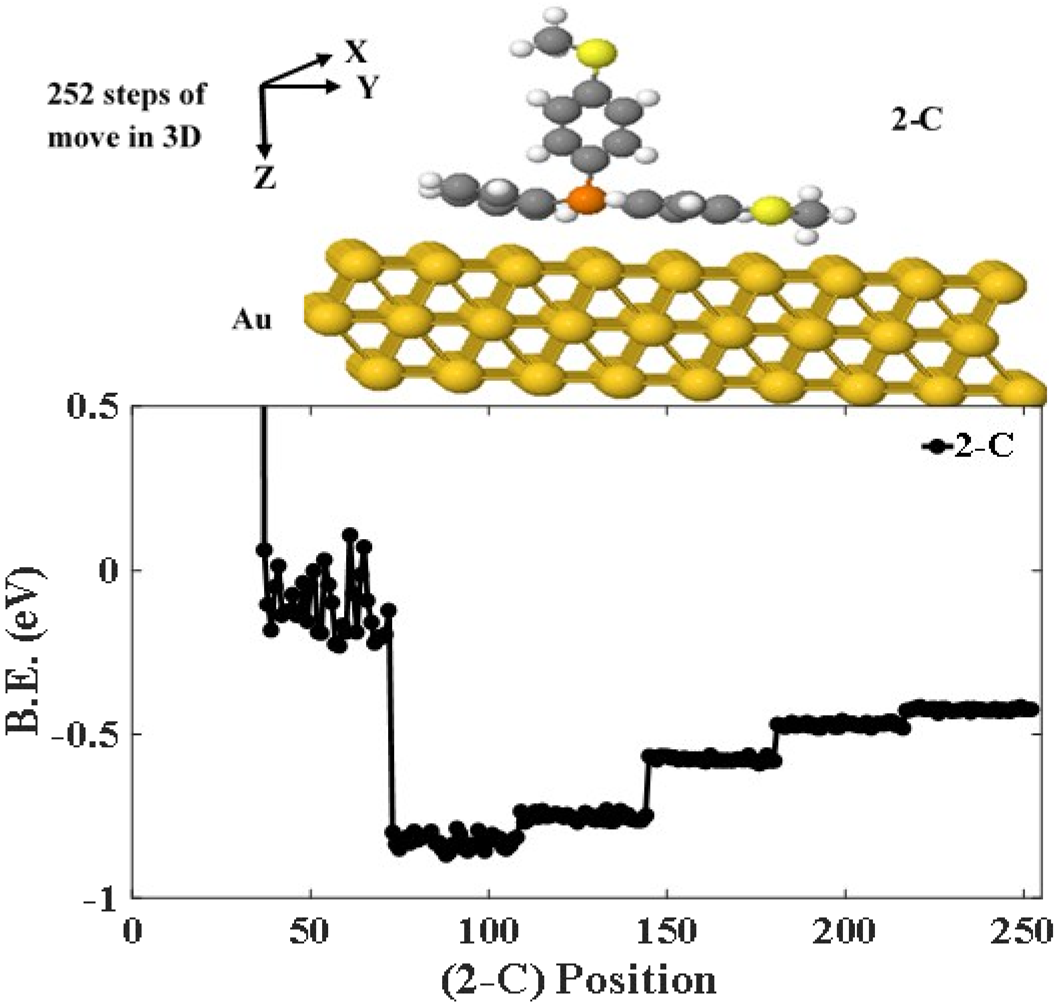

Figure S43: Top panel: Orientation of the **2-C** molecule with respect to gold electrode. **Lower panel**: Binding energy of **2-C** to gold electrode as a function of

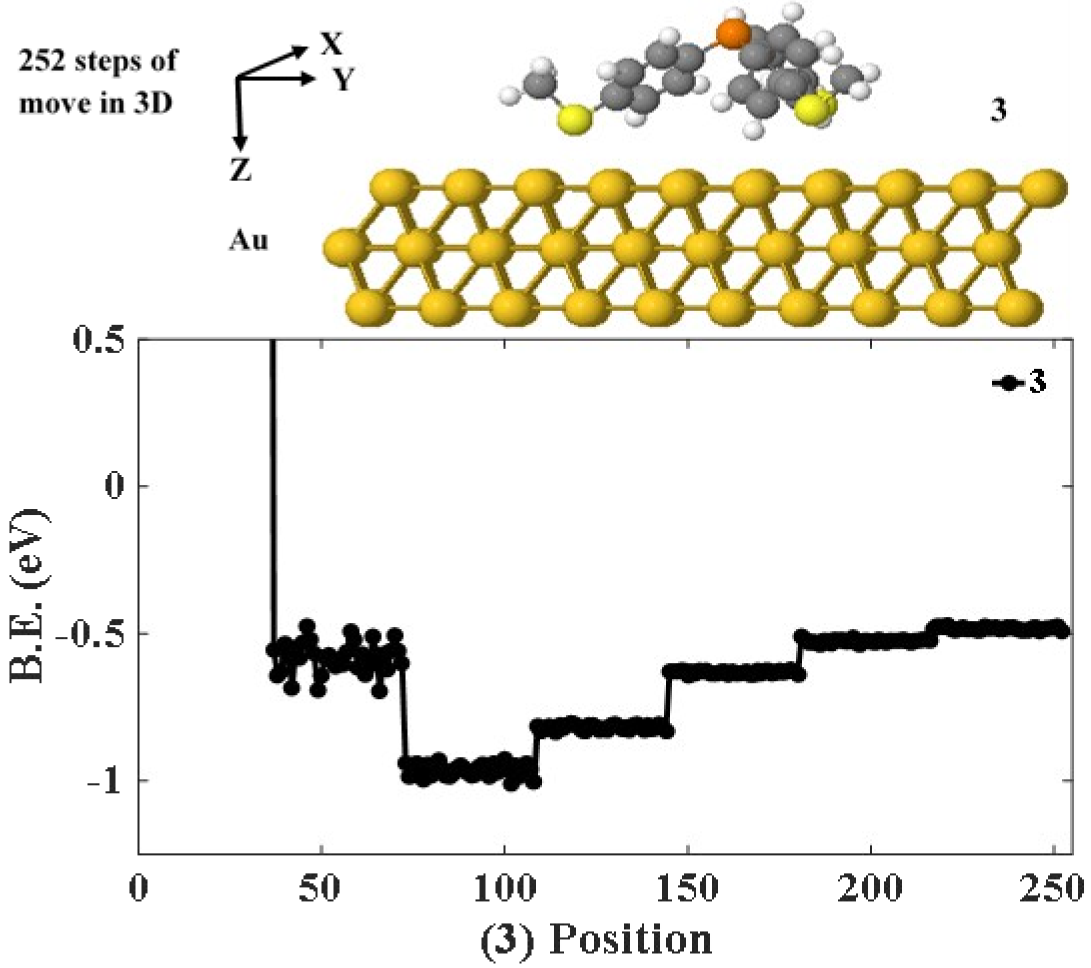

Figure S44: Top panel: Orientation of **3** molecule with respect to gold electrode. **Lower panel**: Binding energy of **3** to gold electrode as a function of position.

3. Conductance

Calculated the conductance's for **3, 3-S** and **3-O** using LDA[2], GGA[3] and Van der Waals[4] functionals. All yield approximately the same results, as shown in Figure S45.

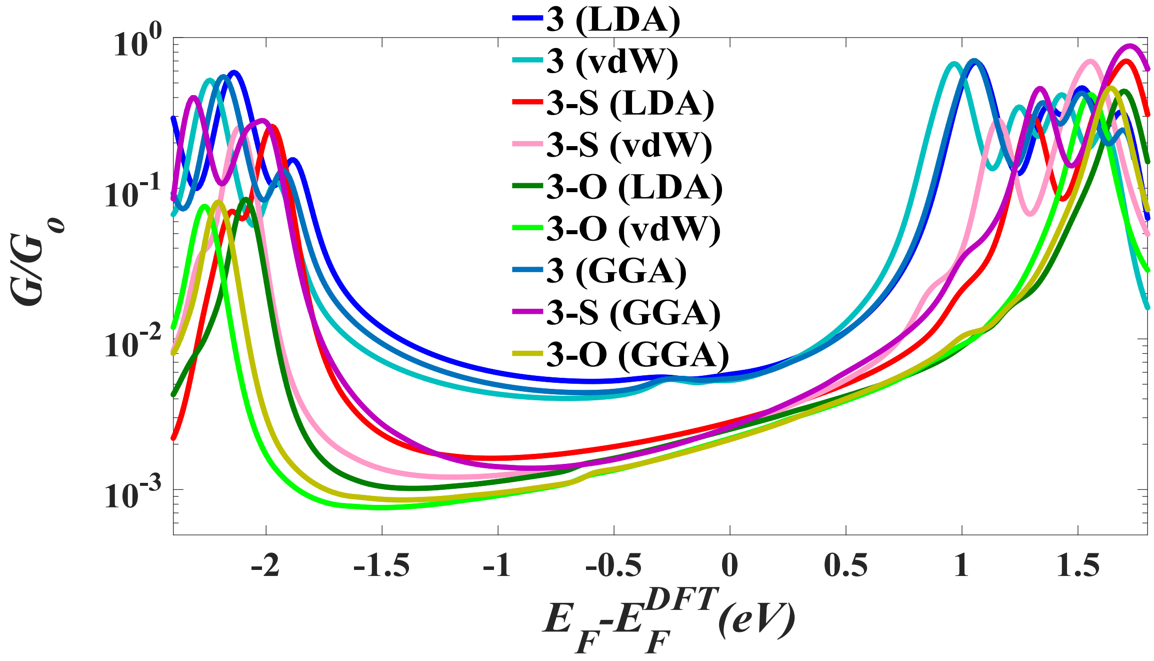

Figure S45. The calculated conductance as a function of the Fermi energy for all molecular junctions, with molecules bound to Au (111) surfaces, obtained using LDA, van der Waals calculations and GGA functionals.

d) References

1. F. Ragaini, L. Lunardi, D. Tomasoni and V. Guglielmi, *J. Organomet. Chem.*, 2004, **689**, 3621-3630.
2. W. Kohn and L. J. Sham, *Phys. Rev.*, 1965, **140**, A1133-A1138.
3. J. P. Perdew, K. Burke and M. Ernzerhof, *Phys. Rev. Lett.*, 1996, **77**, 3865-3868.
4. M. Dion, H. Rydberg, E. Schröder, D. C. Langreth and B. I. Lundqvist, *Phys. Rev. Lett.*, 2004, **92**, 246401.
